# Mapping Microvascular Flow via Radon Transform Ultrasound: Technical Advances and Pilot Application

**DOI:** 10.34133/bmef.0234

**Published:** 2026-02-24

**Authors:** Jingyi Yin, Lijie Huang, Jingke Zhang, U-Wai Lok, Ryan M. DeRuiter, Xiang-yang Zhu, James D. Krier, Yohan Kim, Yanzhe Zhao, Kaipeng Ji, Fabrice Lucien, Lilach O. Lerman, Shigao Chen, Chengwu Huang

**Affiliations:** ^1^ Department of Radiology, Mayo Clinic College of Medicine and Science, Rochester, MN 55905, USA.; ^2^ Division of Nephrology and Hypertension, Mayo Clinic, Rochester, MN 55905, USA.; ^3^ Division of Urology, Mayo Clinic, Rochester, MN 55905, USA.

## Abstract

**Objective:** This study aims to develop a contrast-free, high-sensitivity ultrasound method, denoted as Radon transform-based flow measurement (R-Flow), for in vivo mapping of microvascular flow vectors and for establishing R-Flow-derived vector-field metrics to noninvasively quantify microcirculatory patterns in liver cirrhosis. **Impact Statement:** R-Flow enables robust, contrast-free imaging of microvascular dynamics and demonstrates translational feasibility in the human liver. Its direction-aware indices offer pilot in vivo quantification of flow redistribution and remodeling, providing unique insights into hepatic flow dynamics. **Introduction:** Microvascular dysfunction is a hallmark of many diseases, yet noninvasive visualization and quantitative assessment of abnormal microcirculation remain limited. **Methods:** R-Flow leverages Radon transform to decode red blood cell dynamics from the spatiotemporal domain of ultrasound flow signals, reconstructing velocity vectors at microvascular scale. From these vector maps, unique direction-aware indices are further derived to characterize flow distribution and heterogeneity. **Results:** Validated across simulations, phantoms, and in vivo studies, R-Flow provides robust velocity estimation across a wide range (1 to 60 mm/s). Notably, it enables high-sensitivity microvascular flow vector mapping of human liver, showing strong agreement with references (*r* > 0.9). In a rat model, these direction-aware indices revealed a shift from healthy multipath perfusion in control livers to directionally biased vascular pattern in cirrhotic livers, demonstrating significant correlation with pathological indicators. **Conclusion:** R-Flow enables noninvasive, contrast-free mapping of microvascular blood flow velocity and offers a promising approach for high-resolution assessment of microvascular flow characteristics.

## Introduction

Abnormalities in microvascular flow, such as velocity fluctuations, perfusion heterogeneity, and locally disturbed patterns, are early indicators across disorders and cancer [1,2]. In liver fibrosis and cirrhosis, microcirculatory remodeling is one of the most important pathophysiological features [3]. Distortion of the hepatic microvasculature increases resistance and drives portal hypertension [4,5], correlates with fibrosis stage and clinical outcomes, and limits reversibility. Given the high global burden of advanced fibrosis and cirrhosis, and a 10-year mortality of 34% to 66% in the Western world [3], timely diagnosis and longitudinal monitoring are imperative. While structural alterations can be demonstrated by histology or ex vivo computed tomography (CT) imaging [4], noninvasive and repeatable approaches for characterizing in vivo microvascular flow dynamics without biopsy or ionizing radiation are still lacking [5]. Moreover, how microvascular flow patterns change in cirrhosis remains poorly understood.

Ultrasound is a preferred modality for blood flow imaging due to its noninvasive, nonionizing, and portable nature, making it well-suited for a variety of clinical settings compared with CT, magnetic resonance imaging (MRI), and positron emission tomography (PET) [6]. Doppler ultrasound is considered first-line for blood flow imaging, enabling efficient velocity measurement. However, conventional Doppler imaging detects frequency shifts induced by flowing red blood cells (RBCs) along the beam, yielding only axial component of the in-plane velocity vector, ignoring the lateral component [7]. It is also relatively insensitive to small vessels [8]. Advanced techniques like ultrafast plane-wave functional ultrasound (fUS) have improved microvascular sensitivity [9,10] but still struggle to recover the lateral flow component. This significant pitfall may lead to angle dependency and quantitative bias [11,12], making accurate velocity measurement challenging in complex or tortuous vessels frequently observed in diseased organs, such as the fibrotic liver.

To overcome these limitations and unlock the full potential of ultrasound in microflow assessment, it is imperative to develop methods that can derive complete, angle-independent velocity vectors (axial + lateral). Existing solutions, including vector Doppler [13,14], transverse-oscillation approaches [15], nonlinear parameter estimation [16], localization-based methods [17,18], and speckle tracking [19–21], can reduce angle and operator dependence and reveal complex blood flow patterns such as turbulence. Nevertheless, performance at the microvascular scale or deep human tissue is often challenged by low contrast and spatial resolution [22]. Super-resolution ultrasound (SRUS) imaging strategies, such as ultrasound localization microscopy (ULM), enable super-resolved mapping of microvascular flow velocities [23–25]. However, the required prolonged acquisition time increases susceptibility to tissue motion in clinical scans, and the requirement for microbubble contrast agents restricts their applicability in some patients [17].

Here, we developed R-Flow (Radon transform-based flow measurement), a contrast-free and angle-independent ultrasound method for microvascular flow velocity mapping. Exploiting the unique physical characteristic that flowing RBCs produce coherent trajectories in spatiotemporal domain [26], R-Flow applies the Radon transform to recover both axial and lateral velocity components, yielding complete 2-dimensional (2D) velocity vectors over a wide field of view without contrast agents. In this study, R-Flow’s performance was validated with numerical simulations and phantom experiments, and across various in vivo measurements in a chick embryo, pig kidney, and human liver using clinical ultrasound probes. Furthermore, we proposed direction-aware indices to quantify microvascular flow patterns and validate their pilot feasibility in a rat liver cirrhosis model. To our knowledge, this is the first in vivo, contrast-free ultrasound assessment of flow distribution and microcirculation reorganization in liver cirrhosis, revealing the shift from efficient, multipath perfusion in healthy liver to a less efficient, directionally biased flow pattern in cirrhotic liver.

## Results

### Simulation and phantom results

**Fig. 1.**
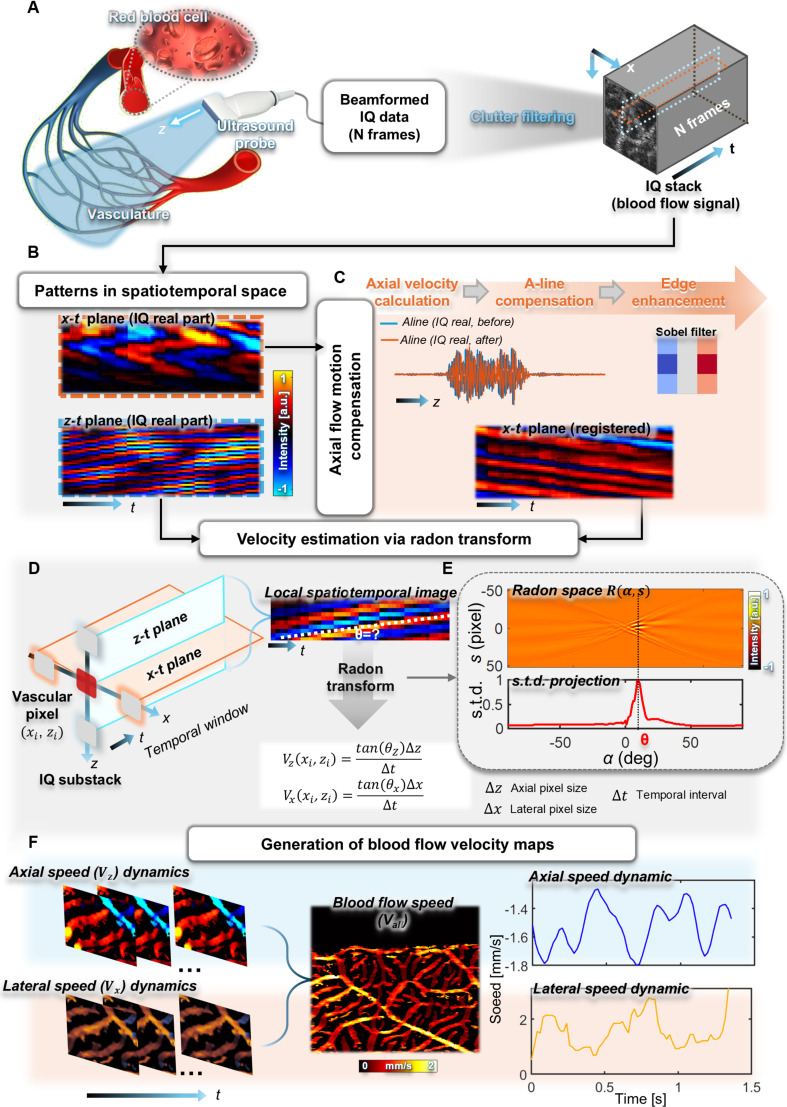
Diagram of R-Flow. Ultrafast plane-wave ultrasound imaging is used to acquire beamformed in-phase and quadrature (IQ) data (A), followed by clutter filtering to extract blood flow signals. Spatiotemporal image slices (*x*–*t* and *z*–*t* planes) are constructed (B and C), from which lateral and axial velocity components are estimated via Radon transform (D and E), respectively. For lateral flow estimation, axial flow motion compensation is applied to enhance the continuity of red blood cell trajectories in *x*–*t* plane (C). R-Flow yields blood flow velocity maps, as well as axial and lateral velocity dynamics (F). More details have been provided in Fig. S1 and Supplementary Methods.

The diagram of R-Flow has been shown in Fig. 1 and Fig. S1.

We validated lateral (*V_x_*) and axial (*V_z_*) velocity estimation using Field II plug flow simulations across flow angles (1° to 90°) and speeds (5 to 25 mm/s). To suppress background and spatial edge effects of spatial window operation, a vascular mask was generated from the normalized cross-correlation map of 2 separately compounded in-phase and quadrature (IQ) datasets, and the binarized version were applied to color Doppler images for fair comparison (details in Supplementary Methods; same for all datasets).

Representative results (Fig. S2A and C) show *V_x_*/*V_z_* maps (additional results in Fig. S3) and corresponding spatiotemporal images of IQ signals (Fig. S2B and D), clearly revealing stripe patterns that reflect velocities of moving scatterers. Notably, after axial flow motion compensation and edge enhancement, continuous lateral–temporal speckle trajectories were revealed (Fig. S2B), even under highly axial-dominant flow conditions (Figs. S4 and S5), enabling robust *V_x_* estimation. Summary statistics (mean and standard deviation of measured velocities within vascular mask) closely follow ground truth across all simulated flow angles (Fig. S2E). Relative error, defined as mean absolute point-wise error normalized by reference measurement (details in Materials and Methods), was ~15% for *V_x_* at lower speeds/small angles and increased with both flow angle and speed (Fig. S2F), whereas *V_z_* remained low, owing to the higher axial resolution of ultrasound imaging. Linear regression analysis of different simulated velocities at a flow angle of 60° yielded slopes of 0.43 for *V_x_* [cos (60°) = 0.50], 0.89 for *V_z_* [sin (60°) = 0.87], and 0.96 for *V_all_*, with coefficients *R*^2^ > 0.99 and *P* < 0.001 (Fig. S2G), confirming the high correlation between R-Flow velocity estimation and ground truth.

Figure S6A to C presents phantom speed maps at set velocities of 0.5, 1, and 4 cm/s, with R-Flow closely matching the reference. Pixel-wise speed map comparisons (Fig. S6D and E) confirm strong agreement with reference measurements, yielding correlation coefficients of *r* = 0.81 to 0.89 for *V_x_* and *r* = 0.91 to 0.96 for *V_z_* (see Fig. S7 for more results). The reference lateral speed values were derived from Doppler-based axial speed estimates combined with the measured tube inclination angle (17°). The speed distributions of R-Flow-estimated *V_x_* and *V_z_* (Fig. S6F and G) follow the expected trends, with higher consistency observed for axial estimates, supporting the feasibility of R-Flow in phantom experiments.

### In vivo validation of R-Flow in chick embryo chorioallantoic membrane

**Fig. 2.**
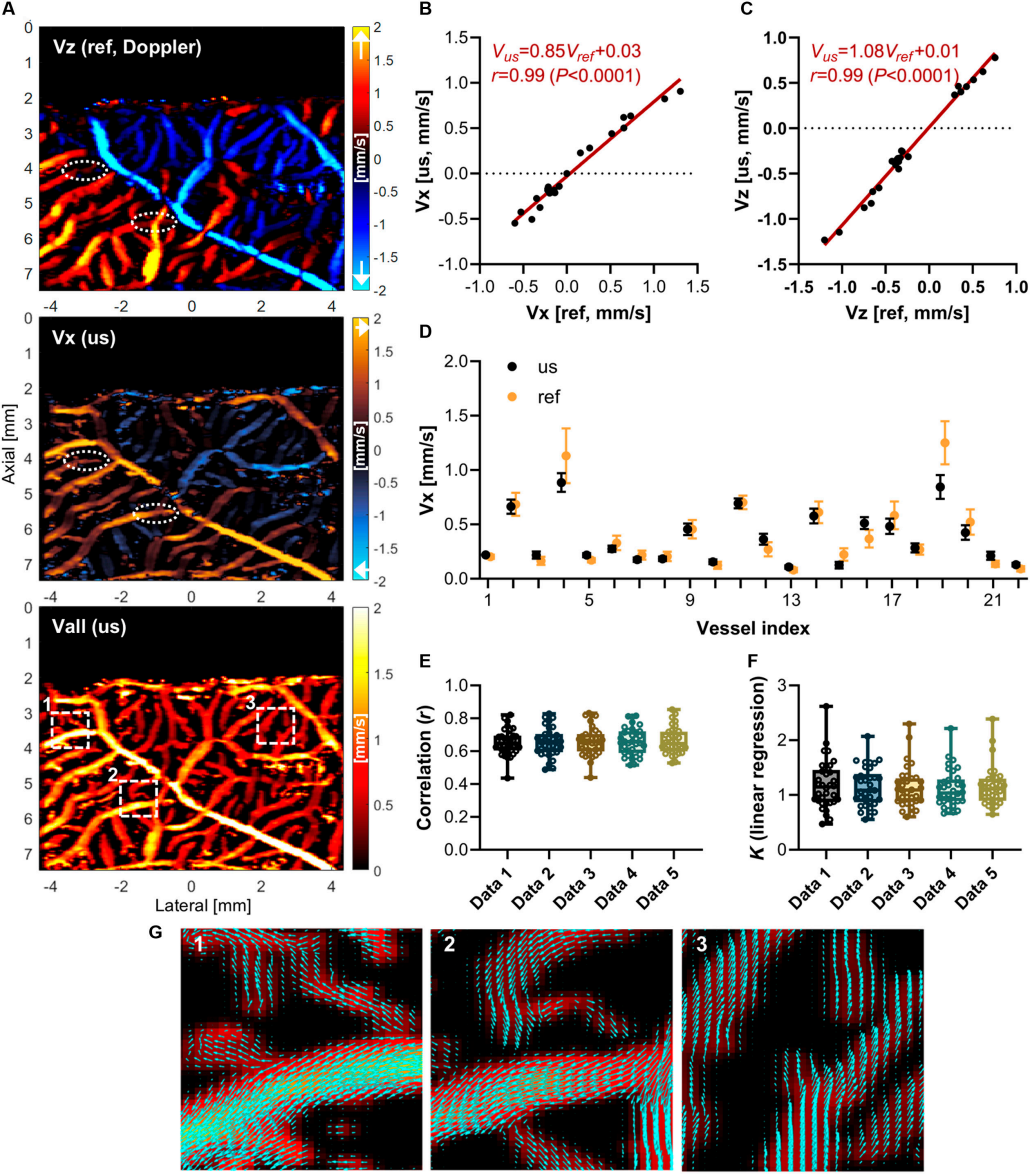
Velocity measurements in chick embryo chorioallantoic membrane. (A) From top: axial velocity map (*V_z_*, ref) obtained using conventional Doppler, lateral velocity map (*V_x_*, us), and the total speed map (*V_all_*, us) measured by R-Flow. (B and C) Scatter plots of the mean lateral (B) and axial (C) velocity from R-Flow versus reference method across 22 vessel regions (locations in Fig. S8). Axial velocity references were obtained from Doppler measurements, while lateral velocities were derived from axial Doppler and measured vessel inclination angles. Linear regression yielded slopes of 0.85 (lateral) and 1.08 (axial), with strong correlations (*r* = 0.99, *P* < 0.0001) in both cases. (D) Comparison of mean lateral speed and standard deviation in 22 vessel regions between R-Flow (black) and reference method (orange). (E and F) Box plots summarize the pixel-wise correlation coefficients (E) and linear regression slopes (F) of the lateral velocity across 22 vessels, evaluated over 5 independent datasets acquired at the same imaging plane. The correlation coefficients and slopes are calculated separately for each vessel segment. (G) Flow field estimated by R-Flow within representative regions of interest, indicated by white rectangles in (A). The local flow direction and speed are visualized using velocity vectors.

Figure 2A shows the Doppler-derived axial velocity *V_z_* (ref), R-Flow-estimated lateral velocity *V_x_* (us), and total flow speed computed from the vector sum, *V_all_* (us). R-Flow resolves multidirectional flow across the vascular network, including lateral components absent in Doppler (white dashed circles). Velocity vectors in Fig. 2G depict local blood flow distribution. Across 22 vessel segments (locations in Fig. S8), mean *V_x_* and *V_z_* ​from R-Flow closely matched references (Fig. 2B and C): slopes 0.85 (lateral) and 1.08 (axial), with *r* = 0.99 (*P* < 0.0001) for both. Lateral velocity distributions (Fig. 2D) also showed high consistency with reference in most vessel segments, which were computed from Doppler-derived axial velocities and manually measured vessel inclination angles. Repeatability was evaluated over 5 independent acquisitions in the same imaging plane. Pixel-wise correlations and regression slopes for *V_x_* ​(Fig. 2E and F; examples in Fig. S8) indicated robust performance, with average correlation ≈0.65 and slopes near 1, supporting the reliability of R-Flow for in vivo lateral flow quantification.

### Velocity mapping in human liver

**Fig. 3.**
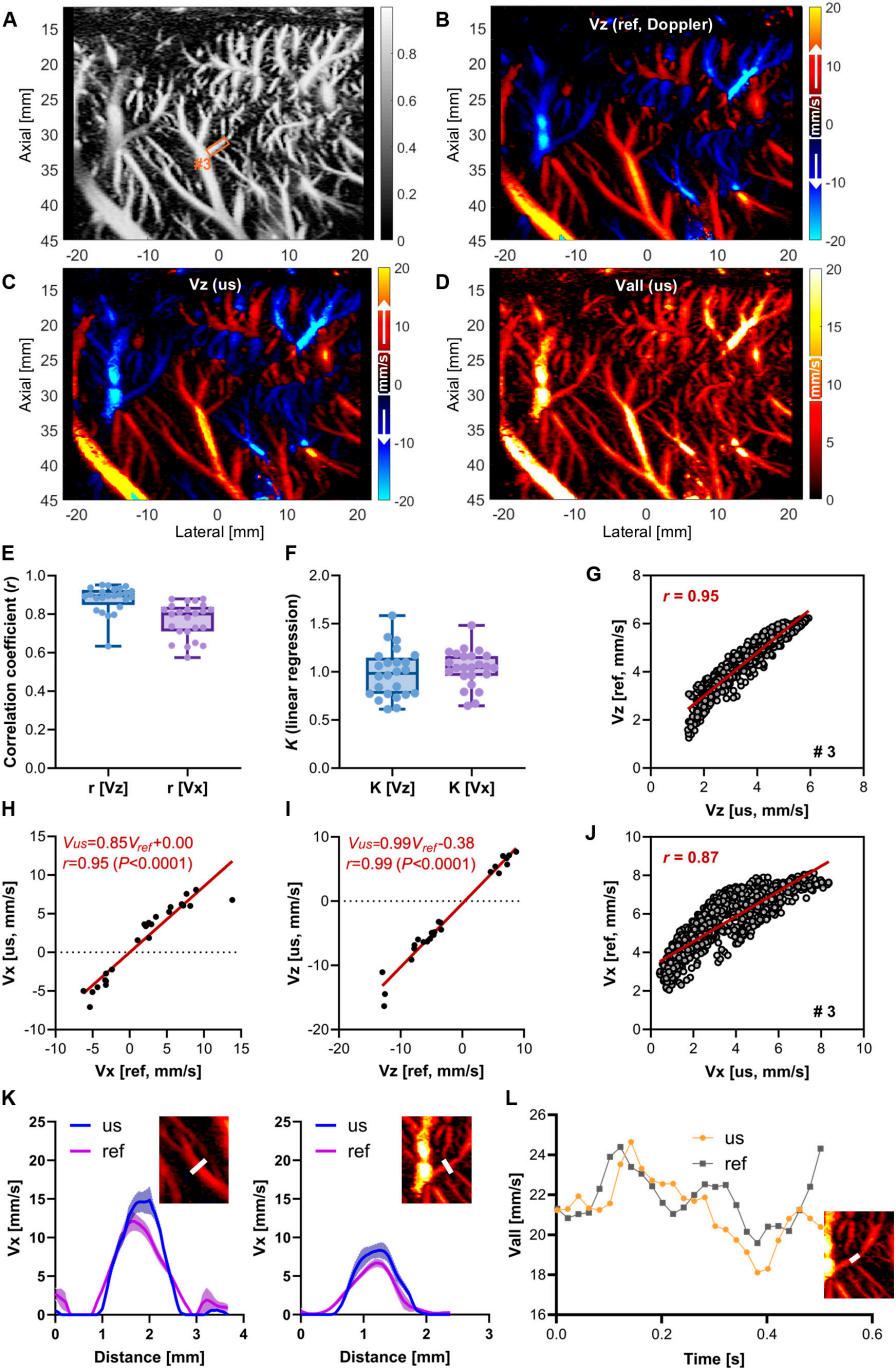
Velocity measurements in a healthy human liver. (A) Vascular mask of liver vasculature. (B to D) Reference axial velocity map (Doppler), R-Flow-estimated axial velocity map *V_z_* (us), and total velocity magnitude *V_all_* (us). (E and F) Box plots of pixelwise correlation coefficients (*r*) and regression slopes (*K*) between R-Flow and reference values across 25 representative vessels (Fig. S7). *r*[*V_z_*] and *r*[*V_x_*] represent the correlation coefficients for axial and lateral velocity components, while *K*[*V_z_*] and *K*[*V_x_*] denote the corresponding regression slopes. Each dot indicates the value from a single vessel segment. Boxes show the interquartile range, and center lines mark the medians. (H and I) Linear regressions of vessel-averaged lateral (H) and axial (I) velocities between R-Flow and reference values across all 25 vessel segments. (G and J) Pixel-wise scatterplots comparing R-Flow-estimated velocities [(G), *V_z_*(us); (J), *V_x_*(us)] and reference values (ref) for vessel #3 [indicated in (A)]. The red line represents the ideal 1:1 fit. (K) Lateral velocity profiles across 2 vessel cross-sections. Shading indicates standard deviation across all measurements over time at each location. (L) Temporal dynamics of mean total flow speed at selected vessel locations, comparing R-Flow (us, orange) and reference measurements (ref, gray).

We further evaluated R-Flow for hepatic microvasculature imaging using a clinical linear array (GE 9L-D). Unlike color Doppler, which measures only axial velocity, R-Flow maps both lateral (*V_x_*), axial (*V_z_*), and full velocity distribution (*V_all_*) (Fig. 3C and D), as illustrated by the flow vector maps in Fig. S9. Across 25 straight vessel segments (locations in Fig. S10), Pearson correlation coefficient *r* and regression slope *K* show strong agreement with references for both velocity components (Fig. 3E and F), with mean slopes ≈1.0 and *r* spanning 0.60 to 0.90. Segment-averaged regression results further confirmed this consistency (Fig. 3H and I), yielding *r* = 0.99 for axial and *r* = 0.95 for lateral velocity. Pixel-wise analyses in a representative vessel showed similar performance (Fig. 3G and J), with *r* = 0.95 (axial) and *r* = 0.87 (lateral). Lateral reference velocities were also derived from Doppler-based axial speed estimates combined with measured vessel inclination. In addition, R-Flow captured lateral speed variations across 2 typical cross-sections (Fig. 3K) and time-varying fluctuations at selected points (Fig. 3L), showing temporal patterns comparable to those measured by color Doppler.

Feasibility at deeper tissues was demonstrated with a clinical curved array in Figs. S11 to S13. Figure S11A to D shows the axial velocity map of color Doppler imaging (*V_z_*), R-Flow-estimated *V_x_*, *V_z_*, and total flow speed (*V_all_*, the vector sum of *V_x_* and *V_z_*). To quantitatively validate R-Flow, we analyzed 28 vessel segments (locations shown in Fig. S12), comparing R-Flow-derived velocities with reference values. Figure S11E and F summarizes the regression slopes (*K*) and correlation coefficients (*r*) for *V_x_* and *V_z_* measurements. The slopes were close to 1.0, and the correlation coefficients were consistently high (e, *r* ≈ 0.8), indicating strong agreement with the reference method. Figure S11H and I presents regression analyses of vessel segment-averaged velocity components, demonstrating strong correlations between R-Flow and the reference measurements (*r* = 0.99 for axial, *r* = 0.97 for lateral velocities). In addition, pixel-wise scatterplots from a representative vessel segment (Fig. S11G and J) also show high correlations between R-Flow-estimated velocities and reference values (*r* = 0.7 to 0.85, more results in Fig. S12). Moreover, Fig. S11K presents lateral velocity profiles and its dynamic range across 2 vessel cross-sections, which are comparable to color Doppler imaging. Additional examples are provided in Fig. S13. Figure S11L shows representative temporal dynamics for *V_x_* and *V_z_* at selected vessel locations. Together, these findings support R-Flow as a noninvasive tool for comprehensive microvascular flow mapping in human liver.

### Comparison of R-Flow and ULM in pig kidney microvascular imaging

We further validated R-Flow against ULM in a healthy pig kidney. ULM-derived velocity maps (Fig. S14A and B; details in Materials and Methods) served as the high-resolution reference. Corresponding R-Flow velocity maps (Fig. S14C and D) exhibited clear spatial concordance with ULM results. To account for different spatial resolutions, pixel-wise comparisons were restricted to locations where the axial flow directions were aligned between the 2 methods. The scatter map of pixel-level velocity measurements (Fig. S14E) demonstrated strong agreement (*r* = 0.87), which was further supported by similar velocity distributions in violin (Fig. S14F) and histogram plots (Fig. S14G). Together, these results demonstrate R-Flow’s robustness for microvascular flow quantification relative to the super-resolution benchmark ULM.

### Altered microcirculatory hemodynamics in liver cirrhosis based on direction-aware indices

**Fig. 4.**
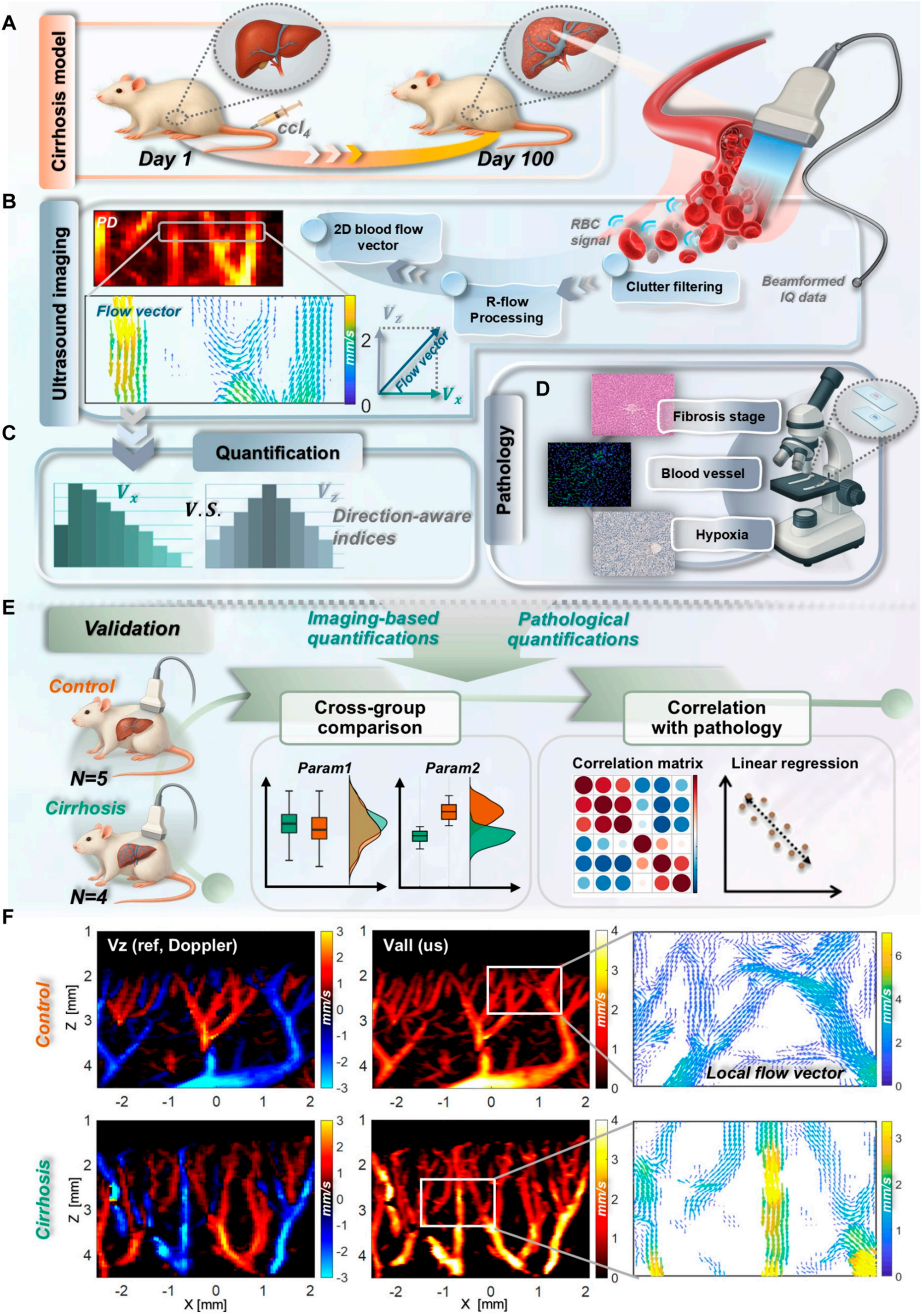
R-Flow vector mapping and validation of direction-aware metrics in a rat model of cirrhosis. (A) Establishment of the CCl_4_-induced rat cirrhosis model. (B) After acquiring beamformed IQ data, blood flow signals were extracted through clutter filtering. R-Flow was then applied to reconstruct the in-plane blood flow vector field based on measured lateral velocity *V_x_* and axial velocity *V_z_*. (C) Direction-aware indices derived from the lateral and axial components (e.g., EFx, SRx, Cx, and DI). (D) Pathology: Quantification of fibrosis stage (Ishak-F score), microvascular density (Dextran), and hypoxia level (Hypoxyprobe). (E) Validation: Cross-group comparisons (control *n* = 5; cirrhosis *n* = 4) and correlations with pathology quantifications (*n* = 8). (F) Representative maps from control and cirrhotic livers: Axial speed (*V_z_*), full speed (*V_all_*), and local flow vectors.

Using a rat model of liver cirrhosis, we applied R-Flow for in vivo microcirculation imaging (Fig. 4A and B). 2D blood flow vector maps were reconstructed by estimating both axial (*V_z_*) and lateral (*V_x_*) velocities (Fig. 4F), and a set of direction-aware indices were computed (Fig. 4C). Pathological analyses were performed (Fig. 4D) and correlated with the proposed indices (Fig. 4E).

**Fig. 5.**
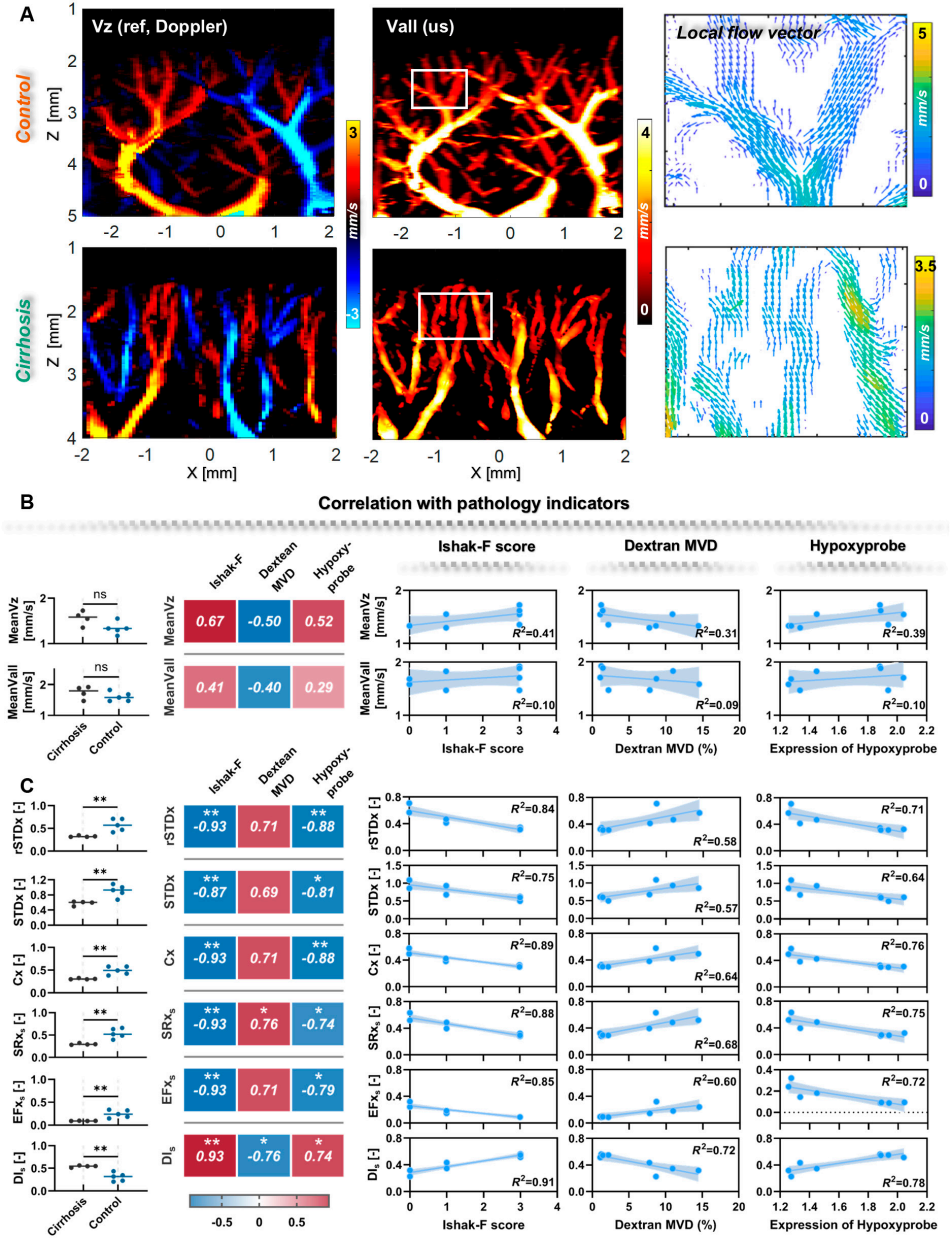
Direction-aware quantification of cirrhosis and control rat livers. (A) Representative maps from control and cirrhosis liver, showing axial speed (*V_z_*, Doppler reference), full speed (*V_all_*), and local in-plane flow vectors. (B and C) Statistics and correlations of traditional speed measurements (B) and direction-aware indices (C). In the left subpanels, each dot represents the metric of a rat. Horizontal bars indicate the group’s mean value. For group comparison, unpaired *t* test was used. The middle heatmaps report Spearman correlation coefficients (*r*, color-coded) between imaging metrics and pathology measurements (*n* = 8), including Ishak-F score, Dextran microvascular density (MVD), and expression of Hypoxyprobe. The right scatterplots provided further details of the relationship between pathological indicators and direction-aware indices. Linear regression was performed with least-squares fits. Shaded bands indicate 95% confidence intervals, and the panels report *R*^2^ values for the linear model. Asterisks mark significance: *P* < 0.05 (*), *P* < 0.01 (**); ns, not significant.

Representative maps from control and cirrhosis liver (Fig. 5A) show axial speed (*V_z_*, Doppler reference), full speed (*V_all_*), and blood flow vectors. As summarized in Fig. 5B, Doppler-derived *V_z_* and R-Flow-derived *V_all_* were similar between groups (*P* > 0.05). In contrast, clear differences emerged in flow distribution: Control livers exhibited a multi-directional, well-distributed perfusion pattern with substantial lateral flow contributions, whereas cirrhotic livers showed channelized, axially biased flow patterns. Quantitatively, direction-aware indices reflecting lateral flow contribution and dispersion were significantly higher in controls (Fig. 5C, all *P* < 0.01), including EFx_s_, rSTDx, STDx, Cx, and SRx_s_, while the axial dominance index (DI_s_) was significantly elevated in cirrhosis (*P* < 0.01). Correlation analysis with pathological indicators further supported these findings (Fig. 5C). All direction-aware indices showed strong correlations with fibrosis stage (Ishak-F score, *r* = 0.87 to 0.93, *P* < 0.01) and hypoxia (Hypoxyprobe, *r* = 0.74 to 0.88, *P* < 0.05). SRx_s_ and DI_s_ were significantly correlated with microvascular density (Dextran MVD, *r* = 0.76 and −0.76, respectively, *P* < 0.05). In contrast, mean *V_z_* and mean *V_all_* showed weak and nonsignificant correlations with pathological markers (*P* > 0.05).

**Fig. 6.**
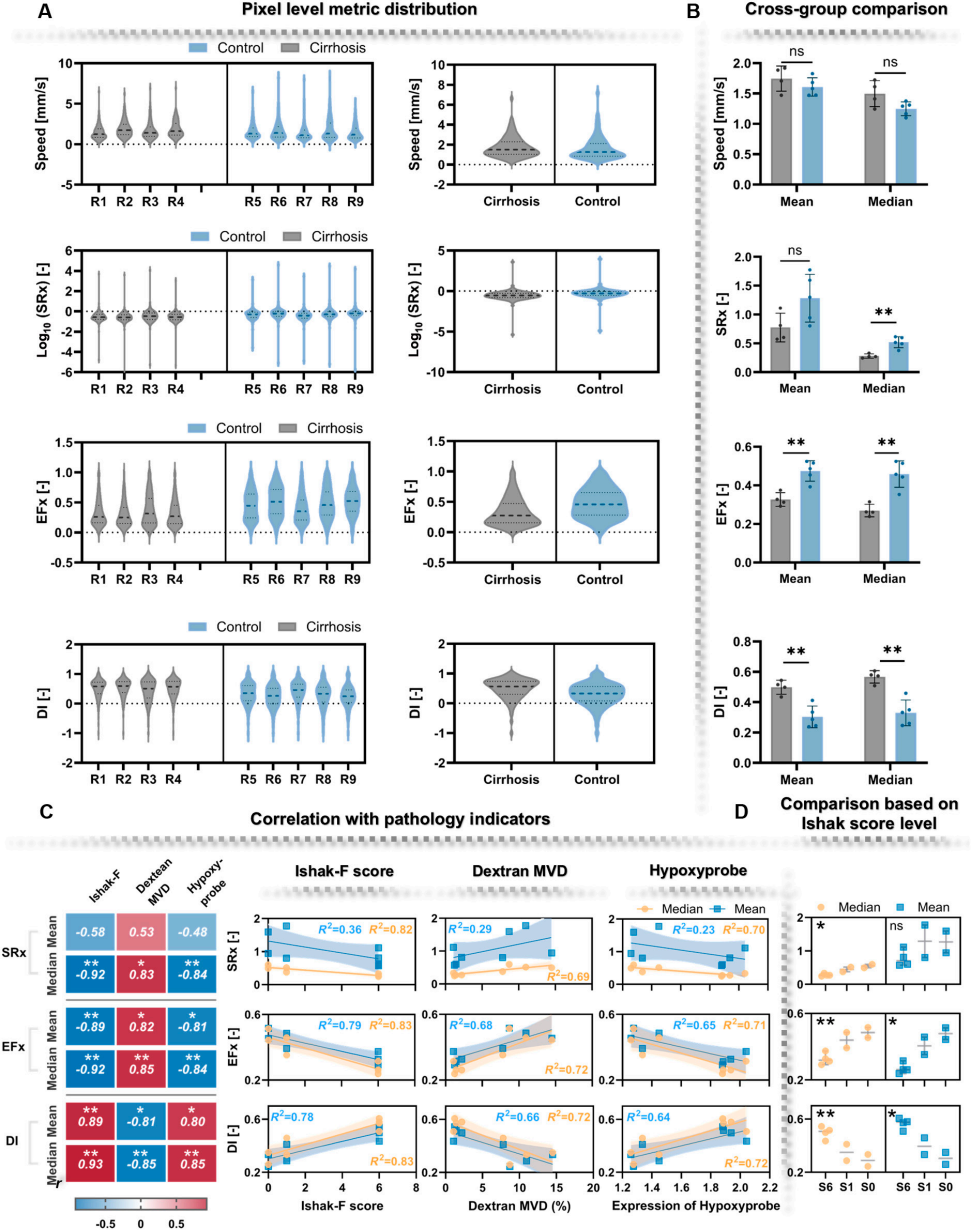
Pixel-level direction-aware metric analysis. (A) Pixel-level distributions. Violin plots show per-animal distributions (left; cirrhosis R1 to R4 in gray, control R5 to R9 in blue) and group summaries (right) of pixel-level direction-aware metrics (EFx, SRx, and DI) and speed values. R1 denotes the rat #1. (B) Between-group comparison. Each dot denotes one animal. Bars show mean ± SD. Asterisks denote statistical significance: ns, not significant; *P* < 0.05 (*); *P* < 0.01 (**). Unpaired 2-sided *t* test with multiplicity adjusted by the Bonferroni–Dunn method. (C) Correlation with pathology. Heatmaps report Spearman’s correlation coefficient (*r*) between imaging metrics and Ishak-F score, Dextran microvascular density (MVD), and expression of Hypoxyprobe (*n* = 8). Right panels show scatterplots with ordinary least-squares fits and 95% confidence bands. (D) Comparison across different Ishak-F stages. Metrics were summarized per animal (median of pixel-level values) and compared across stages using one-way ANOVA with Bonferroni adjustment. S6 denotes Ishak-F = 6.

As shown in Fig. 6B, pixel-level direction-aware metrics were summarized at the subject level using both mean and median values. Both summary strategies yielded similar results: EFx were significantly higher in controls, whereas DI was significantly elevated in cirrhotic livers (all *P* < 0.01), while neither mean nor median flow speed showed significant differences between groups. Strong correlations with pathological measures (Fig. 6C) further supported re-orientation of blood flow in diseased livers. Moreover, both mean and median values of EFx decreased, whereas those of DI increased progressively with advancing Ishak fibrosis stage (Fig. 6D, *P* < 0.05), highlighting the potential of these direction-aware metrics for disease staging.

Taken together, these findings indicate that cirrhosis induces a systematic reorganization of hepatic microcirculation and global flow reorientation that are largely invisible to conventional speed–magnitude measures but are revealed by R-Flow through characterization of flow directionality and distribution. By summarizing velocity vector field into direction-aware indices, we detected a progressive transition from a multipath vascular network (control) to a directionally biased flow pattern (cirrhosis), yielding clearer group separation and stronger correlations with histopathology. These indices may therefore serve as potential biomarkers for early cirrhosis detection and staging, longitudinal treatment monitoring, and assessment of portal hypertension risk.

## Discussion

In this study, we proposed R-Flow, a contrast-free, angle-independent blood flow measurement approach. Unlike conventional Doppler ultrasound, which is inherently limited to detecting axial flow components, R-Flow simultaneously resolves both axial and lateral velocities by analyzing speckle trajectory orientations in the *z*–*t* and *x*–*t* domains, facilitating robust assessment of blood flow perfusion in microvasculature.

In vivo, R-Flow captured microvascular hemodynamics in chick embryo chorioallantoic membrane (CAM; Fig. 2) and human liver (Fig. 3 and Fig. S11), with velocity estimates comparable to reference measurements. By integrating spatiotemporal information, R-Flow maintains robustness across different transducer frequencies and imaging conditions. Unlike speckle-tracking approaches that typically require large spatial windows, R-Flow achieves a practical balance by recovering full velocity vectors from limited temporal frames and moderate spatial window size. Although this introduces a potential trade-off in spatial resolution, the effect is mitigated by vascular pixel identification, which restricts R-Flow analysis to vascular regions. Collectively, these features make R-Flow well suited for visualizing multidirectional microvascular flow in both experimental and clinical settings.

In simulation and phantom experiments, R-Flow achieved higher accuracy in axial velocity (*V_z_*​) estimation, whereas lateral velocity (*V_x_*​) exhibited larger variability, especially at higher speeds and larger vessel angles (Fig. S2). This difference may be primarily attributed to anisotropic spatial resolution in ultrasound imaging: Axial velocity estimation benefits from higher spatial resolution, while lower lateral resolution leads to blurred lateral–temporal speckle trajectories and reduced precision. Two key factors influencing R-Flow performance are the temporal window size ($w_{t}$) and the spatial window sizes ($w_{x}$, $w_{z}$). A larger $w_{t}$​ enhances robustness of trajectory angle estimation but may smear transient flow features, whereas a smaller $w_{t}$​ better tracks rapid changes at the expense of estimation accuracy. Similarly, larger spatial windows ($w_{x}$, $w_{z}$​) help preserve speckle continuity, but may mix spatially distinct flow patterns, while smaller windows may provide insufficient support for reliable speed measurement, particularly for lateral velocity estimation, which is limited by lower spatial resolution. In this study, we constrained spatial windows to 5- to 7-pixel range and temporal windows varied across 50, 100, and 150 frames, providing a practical balance among spatial/temporal resolution and estimation accuracy. Moreover, angular compounding substantially improves the continuity of spatiotemporal trajectories by increasing signal-to-noise ratio (SNR) and suppressing incoherent noise (Fig. S15C to F), thereby enhancing the robustness and reliability of R-Flow. This improvement is more pronounced for lateral flow estimation, which depends strongly on the clarity of lateral–temporal trajectories (Fig. S15G), whereas axial velocity estimation is less sensitive to the number of compounding angles (Fig. S15H).

Moreover, the choice of IQ signal representation directly affects the quality of spatiotemporal trajectories used by R-Flow. For axial velocity estimation, the real and imaginary parts of the IQ signal preserve carrier oscillations, producing dense and coherent streaks in axial–temporal images (Fig. S2D) and resulting in sharp, well-localized peaks in Radon space (Fig. 1E). In contrast, in lateral–temporal images, the real and imaginary components of the IQ signal are strongly influenced by high-frequency phase oscillations originating from axial flow motion (Fig. 1B and Figs. S4 and S5), which obscure the trajectory patterns associated with lateral flow velocity. After axial flow motion compensation and edge enhancement, the IQ envelope yields coherent trajectories in *x*–*t* images (Figs. S4 and S5), leading to improved lateral speed estimation.

For axial speed estimation, as shown in Fig. S16, R-Flow exploits similar phase shift information as conventional color Doppler imaging (lag-one autocorrelation) but estimates velocity from spatiotemporal trajectory patterns in *z*–*t* images rather than pixel-wise phase differences. By integrating coherent flow motion over space and time, R-Flow may improve the robustness of velocity estimation under low-SNR conditions. Similar advantages of trajectory-based axial velocity estimation have also been reported in previous studies [27]. A more comprehensive investigation will be required in future work to compare R-Flow and conventional color Doppler under more complex in vivo conditions.

R-Flow, together with direction-aware indices, is demonstrated as a novel approach for detecting disease-related alterations in hepatic blood flow, providing additional information beyond conventional flow magnitude metrics. Compared to prior reports using phase-contrast CT imaging [5], which focus on vascular structural quantification, R-Flow exploits backscattered signals from moving RBCs to highlight functionally perfused microvessels. It additionally recovers multidirectional, high-resolution flow vector field that is not accessible through standard color Doppler imaging. Importantly, a paradigm shift from the complex, multipath perfusion of the healthy liver to the axially dominated flow distribution of cirrhosis was revealed by the proposed direction-aware indices. In healthy liver, efficient perfusion involves substantial contributions from both lateral (transversal) and axial flow components, resulting in higher metrics that characterize flow balance and dispersion (SRx, rSTDx, STDx, EFx, and Cx), together with a lower axial dominance index (DI). Conversely, progressive accumulation of fibrotic tissue and collagen accumulation in cirrhosis liver may obliterate or narrow substantial portions of microvasculature [3], forcing blood to abandon diverse pathways and flow along the remaining tortuous vessels [4,5]. The loss of efficient, multipath blood flow perfusion introduces a pronounced imbalance between lateral and axial flow distribution, reflected by decreased SRx, rSTDx, STDx, EFx, and Cx, as well as increased DI/DI_s_. Such flow reorientation likely contributes to elevated intrahepatic resistance and the development of portal hypertension [4,5]. The stronger correlation between the direction-aware metrics and Ishak-F score, compared with MVD or Hypoxyprobe, potentially reflects that these metrics more directly capture fibrosis-associated vascular remodeling and flow redistribution [3–5], rather than vessel density or tissue oxygenation. Moreover, despite the modest sample size (Fig. 6D), multiple indices (EFx, SRx, DI) exhibit progressive changes with advancing Ishak fibrosis stage, supporting its potential for disease staging. From the clinical perspective, summarizing the complex velocity field into a single quantitative metric via the proposed direction-aware indices could enable a more objective and operator-independent assessment of blood flow. Importantly, the application of R-Flow and the derived direction-aware indices is not limited to hepatic vascular imaging. R-Flow can be extended to blood flow imaging in other organs, such as the assessment of arteriovenous fistulas in patients with renal disease [28], where multidirectional flow characterization may be clinically relevant. Future patient studies will be required to statistically prove the clinical relevance of the proposed indices.

Despite its strengths, R-Flow has limitations. First, clutter filtering may suppress very slow flows, yielding sparse or weak speckle patterns. Second, the Radon transform presumes linear trajectory continuity, which may not hold in regions with abrupt flow changes or low SNR, potentially affecting estimation accuracy. Third, the current implementation uses 2D acquisitions from 1D arrays and therefore provides a 2D projection of 3D flow. Even so, the recovered in-plane vectors offer richer information than conventional color Doppler imaging. Based on 2D matrix transducers, full volumetric velocity estimation via vector Doppler [29], lateral oscillation [30], and speckle-tracking methods [31] is becoming feasible, which could enable a 3D extension of R-Flow. Fourth, in vivo validation of lateral measurements relies on velocities inferred from Doppler-derived axial speed measurements and calculated vessel inclination angle, which requires straight, clean segments and limits our evaluation in highly tortuous vasculature. Meanwhile, R-Flow’s spatial resolution is limited compared with ULM, which attains super-resolution velocity mapping based on microbubble tracking [23,24], but ULM also demands long acquisitions and contrast injection [8,25]. Finally, future clinical studies are needed to further validate R-Flow’s translational applicability across diverse patient populations.

Several strategies can be adopted to further accelerate R-Flow processing. First, trajectory angles are currently estimated by performing Radon projections of spatiotemporal images over an angular range of 0° to 180° with a 0.5° step size, ensuring accurate estimation of spatiotemporal trajectory orientations. This operation can be replaced by faster alternatives, such as frequency-domain ridge detection, or by adopting a coarse-to-fine strategy in which Radon projections are evaluated only within a narrow angular range around an initial estimate. Second, a major computational burden arises from axial flow motion compensation, which is implemented through repeated 1D interpolation along the axial direction for each frame within multiple spatiotemporal windows. Since these window-based operations are parallelizable, substantial speedups are expected through graphics processing unit (GPU)-based implementations.

In this study, we present R-Flow, a contrast-free ultrasound method that recovers in-plane (axial and lateral) microvascular blood flow velocities by applying the Radon transform to the spatiotemporal trajectories of moving RBC scatterers. Across simulations and phantom tests, R-Flow showed robust performance over 1 to 60 mm/s and captured multidirectional flow with strong correlations to references in human liver and pig kidney. The derived direction-aware indices allow for quantitative assessment of complex microcirculation that are obscured in conventional Doppler ultrasound. To our knowledge, this is the first in vivo, contrast-free ultrasound quantification of flow reorientation and microcirculation remodeling in a rat model of liver cirrhosis, providing unique insights into flow dynamic changes of hepatic vasculature. With its ability to resolve blood flow velocity map in deep human tissue without contrast agents, R-Flow has broader translational potential for early diagnosis and dynamic monitoring of therapeutic response in microvascular-related diseases.

## Materials and Methods

### Technical design of R-Flow

The workflow of R-Flow comprises beamformed IQ acquisition, clutter filtering, axial flow motion compensation, and Radon transform-based velocity estimation. The diagram has been shown in Fig. 1, with details in Supplementary Methods. High-frame-rate ultrasound imaging was used, and spatiotemporal beamformed IQ data $\mathrm{IQ}\left(x,z,t\right)$ were acquired, as shown in Fig. 1A. A localized spatiotemporal clutter filtering approach was adopted to extract blood flow signals from the compounded datasets. Specifically, instead of hard splitting the data into small blocks, 8 × 8 localized data subsets were generated by weighting the original data using Gaussian spatial windows centered at different 8 × 8 grid positions. Eigenvalue decomposition was then performed using the full temporal ensemble for each subset, and tissue clutter was separated from blood flow based on the slope of the eigenvalue curve using a slope threshold of 1.1. The resulting power Doppler images from all subsets were combined by weighted summation, with weights determined by the mean flow signal power of each subset [32]. After clutter filtering, a vascular mask was generated to confine pixel-wise flow estimation and reduce edge effects [33].

In *x*–*t* (lateral–temporal) and *z*–*t* (axial–temporal) slices, the slope of spatiotemporal trajectories encodes the displacement of scatterers over time (Fig. 1B). Accordingly, axial and lateral velocities can be obtained from the tangent of the trajectory angles scaled by the corresponding spatial and temporal sampling intervals. However, the real and imaginary components of the IQ signal in *x*–*t* images are strongly influenced by high-frequency phase oscillations originating from axial flow motion, which obscure the lateral flow patterns (Fig. 1B and Figs. S4 and S5). Therefore, prior to lateral velocity estimation, frame-by-frame axial velocities were estimated and used to compensate for axial flow motion by registering each A-line along the axial direction (Fig. 1C). Subsequently, a 3 × 3 Sobel filter was applied to the compensated IQ envelope to enhance trajectory edges and improve Radon transform-based angle estimation of trajectories in *x–t* images (Fig. 1C).

For each vascular pixel $\left(x_{i},z_{i}\right)$, a sliding spatiotemporal window with spatial width $w_{x}$ (unit: pixel, lateral direction) or $w_{z}$ (axial direction), and temporal length $w_{t}$​ (unit: frame), was used to extract a 2D spatiotemporal image (Fig. 1D). The Radon transform $R\left(\alpha ,s\right)$ was computed for each of the 2D spatiotemporal images (Fig. 1E), where each column represents the projection of the spatiotemporal image ​along angle $\alpha_{k}$​. The trajectory angle θ is identified as the angle $\alpha_{k}$ that maximizes the standard deviation of radon space $R\left(\alpha ,s\right)$ along spatial axis s (vertical dashed line in Fig. 1E). Specifically, based on estimated trajectory angle $\theta_{z}$ from local *z*–*t* image and $\theta_{x}$ from local *x*–*t* image, together with the temporal interval ∆t, pixel sizes ∆x (lateral) and ∆z (axial), the axial and lateral velocity components were calculated as:

$$v_{z}\left(x_{i},\;z_{i}\right)=\frac{\text{tan}\left(\theta_{z}\right)\cdot ∆z}{∆t}$$ (1)

$$v_{x}\left(x_{i},\;z_{i}\right)=\frac{\text{tan}\left(\theta_{x}\right)\cdot ∆x}{∆t}$$ (2)

The total flow speed can be calculated as:

$$v_{\mathrm{all}}\left(x_{i},\;z_{i}\right)=\sqrt{v_{x}{\left(x_{i},\;z_{i}\right)}^{2}+v_{z}{\left(x_{i},\;z_{i}\right)}^{2}}$$ (3)

Dynamic blood flow vector maps were generated by applying computation within a sliding temporal window of length $w_{t}$ (Fig. 1F). The final velocity maps are computed as the mean velocity across all sliding windows. For comparison, conventional Doppler velocities were estimated with the lag-one autocorrelation phase method [34]. All processing was performed in Matlab R2022b (The MathWorks Inc., Natick, MA, USA).

### Subjects and study approval

The in vivo human study was approved by the Institutional Review Board (IRB) at Mayo Clinic. The pig study was approved by the Mayo Clinic Institutional Animal Care and Use Committee (IACUC). All chick embryo experiments were conducted in accordance with institutional guidelines and complied with the United States Public Health Service (PHS) Policy. Since avian embryos are not considered to be live vertebrate animals according to the National Institutes of Health (NIH) PHS policy, no IACUC approval was necessary to perform the chicken embryo experiments in this study. The rat liver dataset was derived from our previously published work [35]. Experimental procedures were conducted in accordance with approved ethical guidelines, with full details reported by Zhang et al. [35].

### Experiment design for R-Flow validation

#### Simulation settings

In this study, tubes with a radius of 2 mm and varying flow configurations were simulated using Field II software [36]. Point scatterers were initially distributed randomly within the pre-set tube area with a density of 20 scatterers per resolution cell. Their positions were updated over time according to predefined uniform flow velocities and flow angles, under a plane-wave data acquisition at a frame rate of 500 Hz. The flow directions were set from the left to the right of the imaging plane. To evaluate the performance of the proposed method under varying flow conditions, 5 different flow speeds (5, 10, 15, 20, and 25 mm/s) and 10 flow inclination angles (1°, and from 10° to 90° in 10° increments) were simulated. Simulation parameters were listed in Table S1. For R-Flow analysis, the spatial window width is set as 5 pixels for lateral direction and 7 pixels for axial direction. The temporal window is 100 frames.

#### Phantom study and ultrasound imaging

Blood-mimicking fluid (Gammex, Middleton, WI, USA) was pumped through a custom-made flow channel phantom (Gammex Inc., Middleton, WI, USA; inner diameter = 2 mm; angled 17° downward) using a motorized syringe pump (NE-1010, New Era Pump Systems Inc., Farmingdale, NY, USA) at mean flow speeds of 0.1, 0.5, 1, 1.5, 2, 2.5, 3, 3.5, 4, 4.5, 5, 5.5, and 6 cm/s. The blood-mimicking fluid flows downward in the axial direction. L11-4v (Verasonics) was used for data collection. A total of 8 diverging waves were transmitted with 2° increments at a center frequency of 4.5 MHz and a post-compounded frame rate of 1,000 Hz. A total of 1,200 frames were collected. The stored IQ data provided lateral and axial pixel sizes of approximately a wavelength (λ ≈ 0.34 mm) and 0.13λ (≈0.045 mm), respectively. The reference lateral speed map (Fig. S6) was estimated based on the axial speed map (Doppler) and the measured tube angle. For R-Flow analysis, the spatial window width is set as 5 pixels for both lateral and axial directions, and the temporal window is 150 frames.

#### Chicken embryo CAM model

In vivo chick embryo CAM is an ideal microvascular model owing to its optical transparency, orderly vasculature, and minimal motion. Ultrasound imaging of chicken embryo vasculature was performed using an L35-16vX linear array transducer (Verasonics Inc., Kirkland, WA, USA) operating at a center frequency of 25 MHz, and the transmit voltage was set as 40 V (one-sided voltage). Fifteen-angle compounded plane wave transmissions were employed, covering an angular range from −7° to +7° with 1° increments. After coherent compounding, the effective frame rate is 500 Hz. The transducer was placed on the side of chick embryo container, allowing lateral access to the surface of the CAM via an acoustic window. Five datasets were acquired in total, each with a duration of 1.5 s, corresponding to 750 frames per acquisition. The final beamformed IQ provided a lateral and axial pixel sizes of 0.05 and 0.02 mm. For R-Flow analysis, the spatial window width is set as 5 pixels for both lateral and axial directions, and the temporal window is 50 frames.

#### Human liver

Ultrasound imaging of healthy human liver was performed using a C1-6-D probe (GE Healthcare, Wauwatosa, WI, USA) and 9L-D linear array transducer (GE Healthcare, Wauwatosa, WI, USA). The parameter settings are shown in Table S2. For R-Flow analysis, the spatial window width is set as 5 pixels for both lateral and axial directions, and the temporal window is 50 frames.

#### Pig kidney

In this study, we used a pig model of chronic kidney disease (CKD), and imaging was performed on the contralateral kidney. During data acquisition, the pig was maintained under anesthesia. Details of pig study have been provided in [37]. Ultrasound imaging was performed using a 9L-D linear array transducer (GE Healthcare, Wauwatosa, WI, USA) operating at a center frequency of 5.2 MHz. Ten-angle compounded plane wave transmissions were employed, covering an angular range from −9° to +9° with 2° increments. The effective frame rate reached 1,000 Hz. Prior to microbubble injection, baseline non-contrast-enhanced ultrasound data were acquired with the probe fixed in position. During this acquisition, a transmit one-sided voltage of 50 V was used. A total of 300 compounded frames were collected, with lateral and axial pixel sizes of 0.15 λ (≈0.04 mm). For R-Flow analysis, the spatial window width is set as 5 pixels for both lateral and axial directions, and the temporal window is 50 frames.

Subsequently, with the probe maintained at the same position, contrast-enhanced ultrasound data were acquired after a bolus injections of Definity MB suspension (Lantheus Inc., MA) through the jugular vein catheter, followed by a flush of saline solution [37]. Subsequently, with acquisition initiated at a proper timing identified by real-time microbubble monitoring [37], ultrasound plane wave imaging was performed at a transmit voltage of 10 V. A total of 3,000 compounded frames were collected. The beamformed IQ data with lateral and axial pixel sizes of 0.3λ (≈0.09 mm) were obtained from the Verasonics ultrasound system.

### ULM processing of pig kidney data

To evaluate the in vivo performance of our proposed R-Flow, we conducted ULM flow measurements for pig kidney data. To suppress tissue signals, a localized spatiotemporal clutter filtering approach was applied. The pixel size was further refined by interpolating to 0.15 of the acoustic wavelengths. Microbubble localization was then performed using a radial symmetry (RS) algorithm [38], which identifies the centroid of each bubble based on intensity gradients. Once localized, the trajectories of individual microbubbles were obtained by applying a tracking algorithm (Simpletracker, MathWorks, Natick, MA, USA) [39]. The tracker implements the Hungarian algorithm, also known as the Kuhn–Munkres algorithm. In our implementation, the “max_gap_closing” parameter was set to zero, enforcing strict continuity in trajectories across frames [38]. Trajectories shorter than 10 frames were considered unstable and discarded. Super-resolution vascular images were reconstructed by accumulating hundreds of thousands of interpolated trajectories. The interframe displacement of microbubble centroids was used to calculate flow velocity. By averaging trajectory speeds at each pixel, super-resolution velocity maps were generated.

### Validation of direction-aware indices in liver cirrhosis model

#### Animal model, image acquisition, and histological evaluation

The data for this section were from a previously published study [35] and were reanalyzed using the novel R-Flow introduced here to demonstrate the added value of the proposed direction-aware indices, which prior methods could not provide [35]. Male Sprague–Dawley rats (200 to 250 g) were maintained under controlled conditions. Cirrhosis was induced in *n* = 4 rats by subcutaneous administration of a 50% carbon tetrachloride solution in olive oil given twice weekly, and a loading dose of 0.6 ml per 100 g body weight was followed by 0.3 ml per 100 g for subsequent injections [35]. Controls (*n* = 5) received no CCl₄. Ultrasound imaging was performed 100 d after the first injection using Vevo 3100 system with a 40-MHz linear array (MX500D, Fujifilm, VisualSonics). The liver was exposed via laparotomy, and after selecting a plane in the right liver lobe, the probe was secured in a holder, and 1,000 B-mode IQ frames were recorded and stored (frame rate: 592 Hz).

Pathological fibrosis, blood perfusion, and hepatic hypoxia analysis of 8 mice was acquired [35]. For histological analysis, liver tissue fixed in 10% neutral-buffered formalin and embedded in paraffin (FFPE) was sectioned and stained with hematoxylin and eosin (H&E) and Sirius Red. Fibrosis stage was assigned blinded by an experienced hepatopathologist (~10 years) using the Ishak scoring system. To visualize vascular space and perfusion, animals received an intravenous fluorescein isothiocyanate (FITC)–dextran injection (50% w/v in saline; Sigma-Aldrich, 500 mg/kg) 10 min before tissue harvest [40]. Fluorescence images were acquired on a Nikon microscope, and vessel density was quantified in ImageJ. For hypoxia mapping, pimonidazole (Hypoxyprobe-1 kit; 60 mg/kg, intraperitoneally) was administered 2 h before ultrasound acquisition and liver collection. Slides were rinsed, counterstained with erythrosine, and mounted (CC/Mount, Sigma), and hypoxyprobe signal was quantified using ImageJ [41]. The vessel density of Dextran and expression of Hypoxyprobe were assessed in 5 successive high-magnification (200×) fields for each rat, and the mean value of these 5 counts was determined. Details have been provided in [35].

#### Direction-aware indices

R-Flow reconstructed a 2D velocity vector field at each vascular pixel: lateral (transversal) velocity *V_x_* ​ along the probe and axial (depth) velocity *V_z_*. From these vectors, we derived direction-aware indices, including:

1. Lateral energy fraction: $\mathrm{EFx}\;=\;\frac{{V_{x}}^{2}}{{V_{x}}^{2}+{V_{z}}^{2}}$, and ${\mathrm{EFx}}_{s}\;=\;\frac{\sum {V_{x}}^{2}}{\sum \left({V_{x}}^{2}+{V_{z}}^{2}\right)}$;
2. Lateral-axial speed ratio: $\mathrm{SRx}=\frac{\left|V_{x}\right|}{\left|V_{z}\right|}$, and ${\mathrm{SRx}}_{s}=\frac{\sum \left|V_{x}\right|}{\sum \left|V_{z}\right|}$;
3. Lateral-axial variability ratio: $\text{rSTDx}=\frac{\mathrm{std}\left(V_{x}\right)}{\mathrm{std}\left(V_{z}\right)}$;
4. Lateral speed dispersion: $\text{STDx}=s\mathrm{td}\left(V_{x}\right)$;
5. Normalized lateral complexity: $\mathrm{Cx}=\frac{\mathrm{std}\left(V_{x}\right)}{\sqrt{\mathrm{std}{\left(V_{x}\right)}^{2}+\mathrm{std}{\left(V_{z}\right)}^{2}}}$;
6. Axial dominance index: $\mathrm{DI}=\frac{\left|V_{z}\right|-\left|V_{x}\right|}{\left|V_{z}\right|+\left|V_{x}\right|}$, and ${\mathrm{DI}}_{s}=$$\frac{\sum \left|V_{z}\right|-\sum \left|V_{x}\right|}{\sum \left|V_{z}\right|+\sum \left|V_{x}\right|}$.

### Statistical analysis

Relative error was calculated to compare the flow velocity measurement results:

$$\text{Relative error}=\frac{1}{\text{NP}}\sum_{i=1}^{\text{NP}}\frac{\left|v_{\mathrm{us}}^{i}-v_{\mathrm{ref}}^{i}\right|}{\left|v_{\mathrm{ref}}^{i}\right|}$$ (4)

where *NP* denotes the number of selected pixels. $v_{\mathrm{us}}^{i}$ and $v_{\mathrm{ref}}^{i}$ are the velocity values at each point measured by R-Flow and the reference method, respectively.

All statistical analyses were performed using GraphPad Prism 10.3 (GraphPad Software, San Diego, CA, USA). Linear regression analysis was used to assess the relationship between estimated and reference velocities, and Pearson correlation coefficient (*r*) was calculated to quantify their correlation. Differences between cirrhotic and control groups were tested with 2-sided *t* tests for normally distributed data and Mann–Whitney *U* tests for non-normal data. Multiple comparisons were corrected using the Bonferroni–Dunn method. Correlations with pathological indicators (Ishak fibrosis score, expression of Hypoxyprobe, Dextran MVD) were measured with Spearman rank correlation. Indices across 3 Ishak fibrosis stages were compared using one-way analysis of variance (ANOVA) with Bonferroni adjustment. A 2-sided *P* < 0.05 was considered statistically significant.

## Data Availability

The data of animal study that support the findings of this study are available upon reasonable request from the corresponding authors. The data of human patients are not publicly available due to privacy or ethical restrictions.
