# Supplementary material for: Mapping Microvascular Flow via Radon Transform Ultrasound: Technical Advances and Pilot Application
Source: BME Front. 2026 Feb 24;7:0234. doi: 10.34133/bmef.0234 (PMC13274627; doi:10.34133/bmef.0234)
Supplement: Supplementary 1 — Supplementary Methods Figs. S1 to S16 Tables S1 and S2 [file bmef.0234.f1.docx]

**Supplementary Materials for**

**Mapping Microvascular Flow via Radon Transform Ultrasound: Technical Advances and Pilot Application**

*Corresponding author. Email: Chen.Shigao@mayo.edu, [Huang.Chengwu@mayo.edu](mailto:Huang.Chengwu@mayo.edu)

The file includes:

Supplementary Method

Supplementary Figures S1 to S16

Supplementary Tables S1 to S2

### **Supplementary Method**

R-Flow estimates blood flow velocity using beamformed IQ data. For each vascular pixel $(x_{i},z_{i})$, spatiotemporal images are constructed along the axial and lateral directions separately to characterize corresponding velocity components. The detailed steps are illustrated below (**Fig. S1**).

1. **Identification of Vascular Pixels**

Given the complex spatial variability of tissue clutter and background noise in large field-of-view imaging, a local clutter filtering strategy was adopted to extract the blood flow signals from the beamformed IQ data [32]. Meanwhile, to suppress incoherent artifacts and background noise, tilted plane/diverging wave acquisitions were divided into two angle sub-groups, yielding two compounded datasets ${IQ}_{1}(x,z,t)$ and ${IQ}_{2}(x,z,t)$. Each sub-group was independently clutter filtered, and the normalized cross-correlation between the two filtered outputs, denoted as ${{IQ}_{1}}^{c}\left( x,z,t \right)$ and ${{IQ}_{2}}^{c}(x,z,t)$, was computed to generate a vascular mask $M(x,z)$, which was subsequently applied to enhance contrast of blood flow signals [32]:

$IQ\left( x,z,t \right)=({{IQ}_{1}}^{c}\left( x,z,t \right)+{{IQ}_{2}}^{c}(x,z,t))\times M(x,z)$ (1)

For rat liver data, all frames of clutter-filtered IQ signals were divided into two temporal sub-groups, and normalized cross-correlation between them was calculated to generate a vascular mask $M(x,z)$. Meanwhile, to enhance efficiency and suppress spatial edge effects induced by spatial window operation, a threshold of $M(x,z)$ was determined through Otsu’s method to identify all vascular pixels. Subsequent R-Flow estimation was limited to the segmented vascular regions. This binary mask was also used to suppress background noise in the color Doppler images.

1. **Estimation of Axial Velocity Components**

The axial velocity is estimated by calculating the inclination angles of streak patterns (trajectory angle) in axial-temporal (*z*-*t*) images. For a pixel at $\left( x_{i},z_{i} \right)$, a spatiotemporal image ${U^{z}}_{(x_{i},z_{i})}$ of size $w_{z}\times w_{t}$​ is extracted using the real component of the $IQ\left( x,z,t \right)$, where:

- $w_{z}$ denotes the number of spatial pixels in the axial direction.
- $w_{t}$ denotes is the number of successive frames.

The inclination angle of the blood flow trajectories in ${U^{z}}_{(x_{i},z_{i})}$ corresponds to the flow direction and magnitude of moving blood flow within that selected spatio-temporal window. Radon transform is employed for angle estimation:

$R(\alpha,s)=\frac{1}{w_{z}w_{t}}\int_{z=1}^{w_{z}} \int_{t=1}^{w_{t}} {U^{z}}_{\left( x_{i},z_{i} \right)}\left( z,t \right)\cdot\delta(s-zcos\alpha-tsin\alpha)dtdz$ (2)

Here, ${U^{z}}_{(x_{i},z_{i})}$ represents the spatiotemporal image, and $\delta$ (⋅) represents the Dirac delta function integrates along projection lines defined by angle $\alpha$ and position $s$. $\Delta z$ represents the axial pixel size, and $\Delta t$ is the acquisition time interval between consecutive frames. The projection coordinate $s$ is defined as the distance from the origin to a line oriented at angle $\alpha$.

By projecting the spatiotemporal image ${U^{z}}_{(x_{i},z_{i})}$ along various angle candidates, denoted as $\alpha_{k} (k=1, 2,\ldots,N)$, the Radon transform maps dominant inclination angle in ${U^{z}}_{(x_{i},z_{i})}$ to a concentrated energy peak in Radon space $R(\alpha,s)$. In this study, we perform Radon transform on $\alpha_{k}$ = [0 180°] with a step size of 0.5° to ensure accurate trajectory angle estimation. The standard deviation of each $\alpha_{k}$ is then calculated along $s$ axis, and the angle corresponding to the maximum standard deviation (indicated by the vertical black dashed line in **Fig. 1**), denoted as $\theta_{z}$, is selected as the estimated trajectory angle. Since the projection at angle $\theta_{z}$ contains the highest image contrast, it is regarded as the optimal estimate of the average trajectory direction within ${U^{z}}_{(x_{i},z_{i})}$. The axial velocity is then computed as:

$v_{z}(x_{i},z_{i})=\frac{\Delta z}{\Delta t}\cdot tan(\theta_{z})$ (3)

The sign of the tangent of the estimated trajectory angle $\theta_{z}$​ is used to determine the flow direction along the axial axis:

$d_{z}(x_{i},z_{i})=sign(tan(\theta_{z}))$ (4)

To ensure robust estimation, a quality metric resembling the signal-to-noise ratio (SNR) is calculated:

$S\left( \theta_{z} \right)=\frac{{Var}_{s}\{R(\theta_{z},s)\}}{\frac{1}{N}\sum_{k=1}^{N} {Var}_{s}\{R(\alpha_{k} ,s)\}}$ (5)

where $\alpha_{k} (k=1, 2,\ldots,N)$ represent the $N$ candidate projection angles used during the Radon transform. ${Var}_{s}\{R(\theta_{z},s)\}$ defines the variation in the Radon image $R(\alpha,s)$ along the direction $s$ at the estimated inclination angle $\theta_{z}$. Only those estimates satisfying $S\left( \theta_{z} \right)>2$ are retained as valid measurements.

1. **Axial Flow Motion Compensation**

The real and imaginary components of the IQ signal in *x*-*t* images are strongly influenced by high-frequency phase oscillations originating from axial flow motion, which obscure the lateral flow patterns (**Fig. S4** and **S5**). To mitigate this effect, axial flow motion compensation is applied by registering each A-line along the axial direction.

Firstly, the frame-by-frame axial velocity $v_{z}(x_{i},z_{i},t_{j})$ is estimated through lag-one autocorrelation phase method [34]. Given the temporal sampling interval $\Delta t$ and the axial pixel size $\Delta z$, the number of axial pixels shifted at every pixel $(x_{i},z_{i},t_{j})$ compared to the first frame in the temporal window (denoted as $t_{s}$) is given by:

$N_{shift}\left( x_{i},z_{i},t_{j} \right)=\frac{\int_{t_{s}}^{t_{j-1}} v_{z}\left( x_{i},z_{i},t_{j-1} \right)\cdot\Delta t}{\Delta z}$ (6)

Then, every A-line is registered according to the estimated axial flow displacement:

${IQ}^{'}(x_{i},z_{i},t_{j})=IQ\left( x_{i},z_{i}-N_{shift}\left( x_{i},z_{i} \right),t_{j} \right)$ (7)

${IQ}^{'}$ is the axial-motion-compensated ultrasound signal. Sub-pixel shifts are implemented using cubic interpolation.

1. **Estimation of Lateral Velocity Components**

Using the compensated IQ signal ${IQ}^{'}(x,z,t)$ , the lateral velocity is estimated similarly. Specifically, we computed the lateral velocity component using Radon transform based on the envelope of ${IQ}^{'}(x,z,t)$. For a selected pixel$\left( x_{i},z_{i} \right)$, a spatiotemporal image ${U^{x}}_{(x_{i},z_{i})}$ of size $w_{x}\times w_{t}$​, is extracted, where $w_{x}$ denotes the number of spatial pixels in the lateral direction, and $w_{t}$ denotes the number of selected frames. To enhance speckle trajectories, we then applied 3-pixel $\times$ 3-pixel vertical Sobel filter on each spatiotemporal image ${U^{x}}_{(x_{i},z_{i})}$. After axial flow motion compensation and edge enhancement, the blood flow trajectories in the lateral-temporal images become more coherent and continuous, as shown in **Fig. S4** and **S5**.

Following the same approach as (2), the trajectory angle $\theta_{x}$ is extracted using Radon transform, and the lateral velocity is calculated as:

$v_{x}(x_{i},z_{i})=\frac{\Delta x}{\Delta t}tan(\theta_{x})$ (8)

$\Delta x$ represents the lateral pixel size and $\Delta t$ is the acquisition time interval between consecutive frames. The sign of the tangent of the estimated inclination angle $\theta_{x}$​ is used to determine the flow direction along the lateral axis:

$d_{x}(x_{i},z_{i})=sign(tan(\theta_{x}))$ (9)

1. **Generation of Blood Flow Velocity Maps**

Steps 2-4 are performed for all vascular pixels. Then, the total flow speed can be calculated as:

$v_{all}(x_{i},z_{i})=\sqrt{{v_{x}(x_{i},z_{i})}^{2}+{v_{z}(x_{i},z_{i})}^{2}}$ (10)

By applying a temporal window of $w_{t}$ frames at a step size of $K (K<w_{t})$ frames, sequences of lateral, axial, and total velocity maps can be generated, enabling dynamic hemodynamic analysis. The final velocity maps are computed as the mean velocity across all sliding windows.

### **Supplementary Figures**


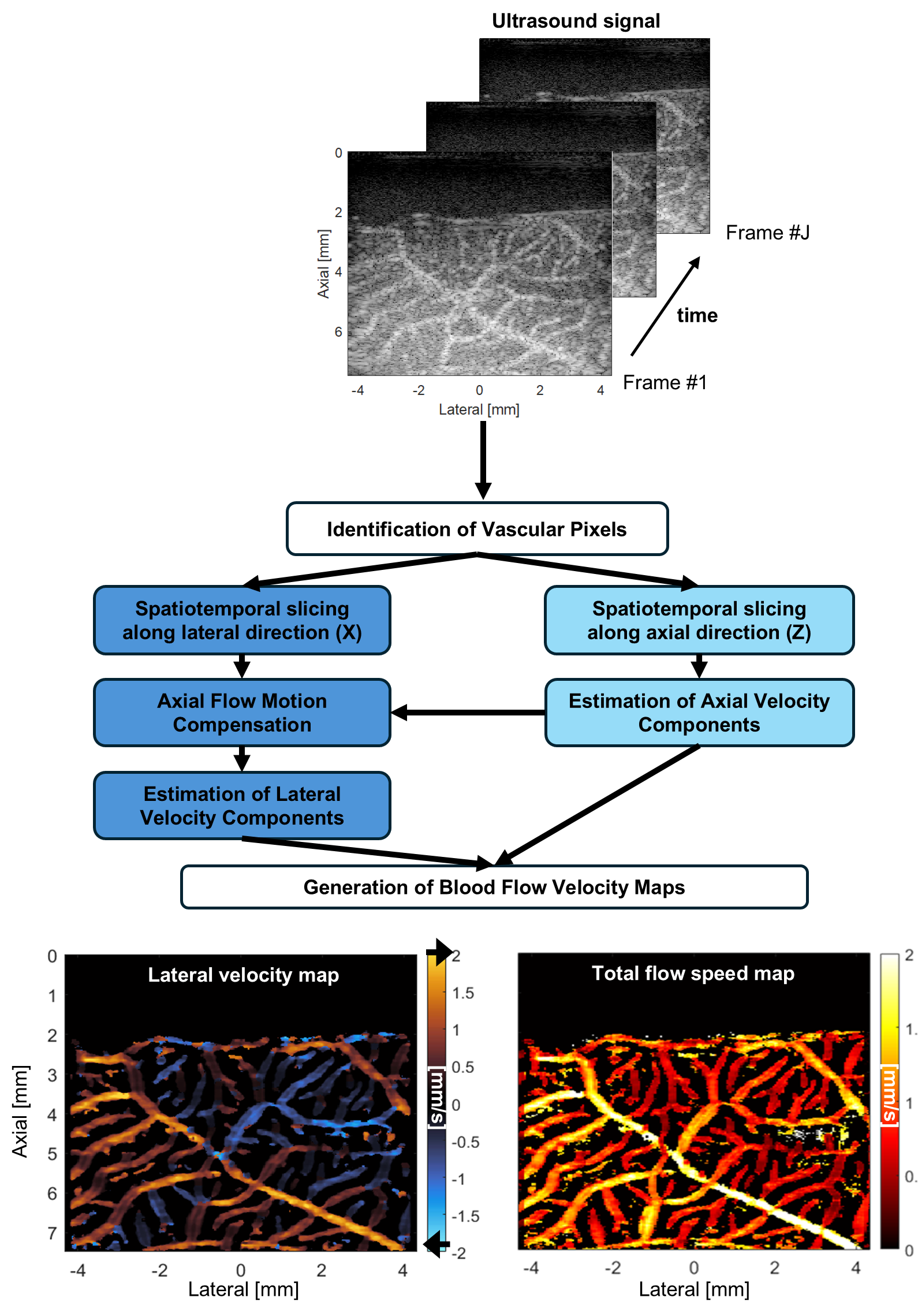


**Fig. S1.** Diagram illustrating the methodological steps of R-Flow.


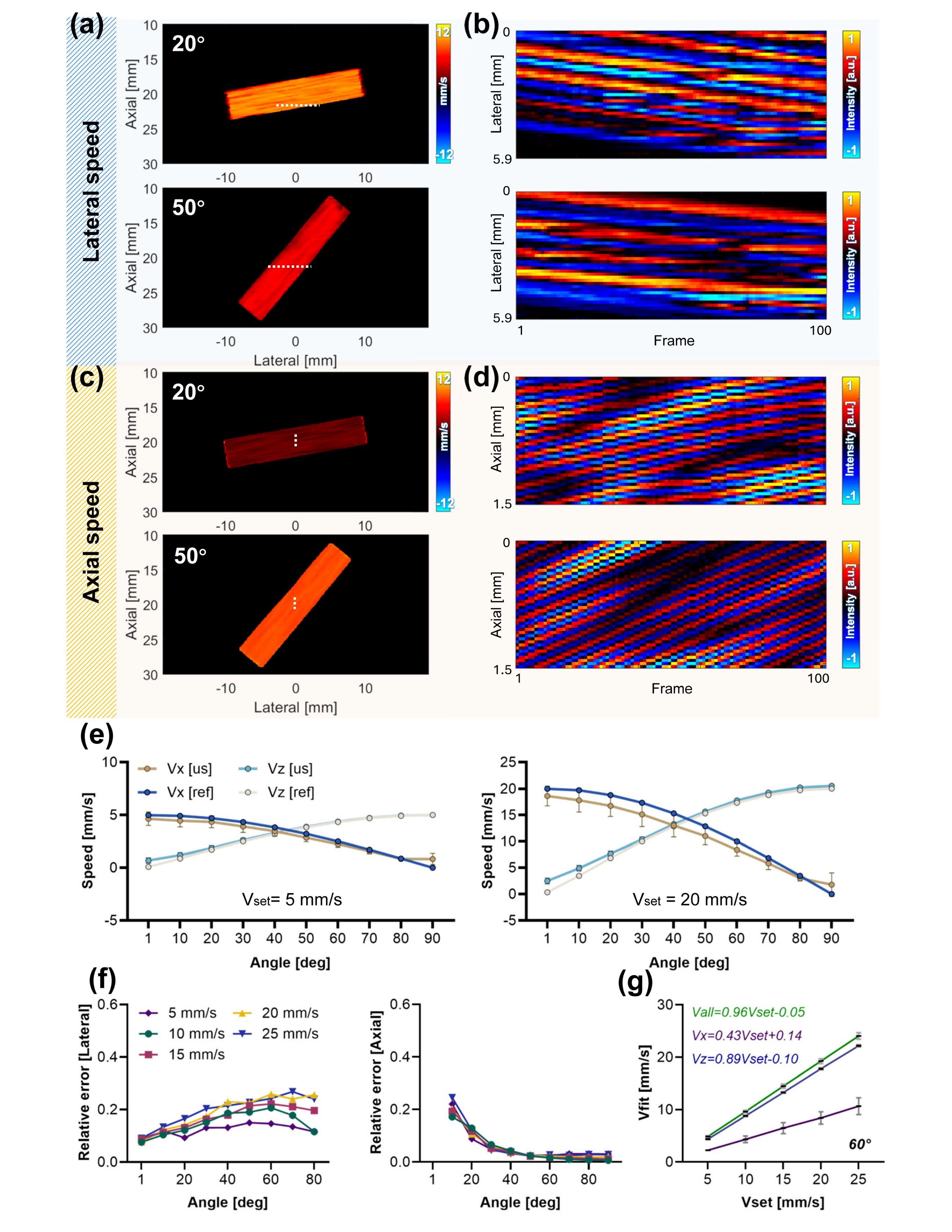


**Fig. S2.** Simulation results of R-Flow. Measured lateral (a) and axial (c) velocity maps under different flow speeds and angles. (b, d) Corresponding spatiotemporal images extracted along the white dashed lines: (b) Lateral-temporal slices showing the envelope of IQ signal after Sobel filter enhancement. (d) Axial-temporal slices showing the real part of the IQ signal. Stripe patterns in the spatiotemporal images represent the spatiotemporal trajectories of moving speckles. (e) Comparison of the estimated lateral (*V_x_*) and axial (*V_z_*) velocities obtained by the proposed method (us) and the corresponding simulated velocities (ref) under various flow angle and velocity settings. Error bars: standard deviation of measured speed values in the simulated tube. (f) Relative errors of *V_x_* and *V_z_* under different preset velocities (5–25 mm/s) and flow angles. (g) The fitted lines obtained from linear regression between the estimated velocities (*V_fit_*) and the preset velocities (*V_set_*) at a flow angle of 60°. The estimated velocities show good consistency with the ground truth, with fitted slopes of 0.43 [cos(60°) = 0.5] for *V_x_*, 0.89 [sin(60°) = 0.87] for *V_z_*, and 0.96 for *V_all_*. Error bars (gray): standard deviation of measured speed values in the simulated tube.

**
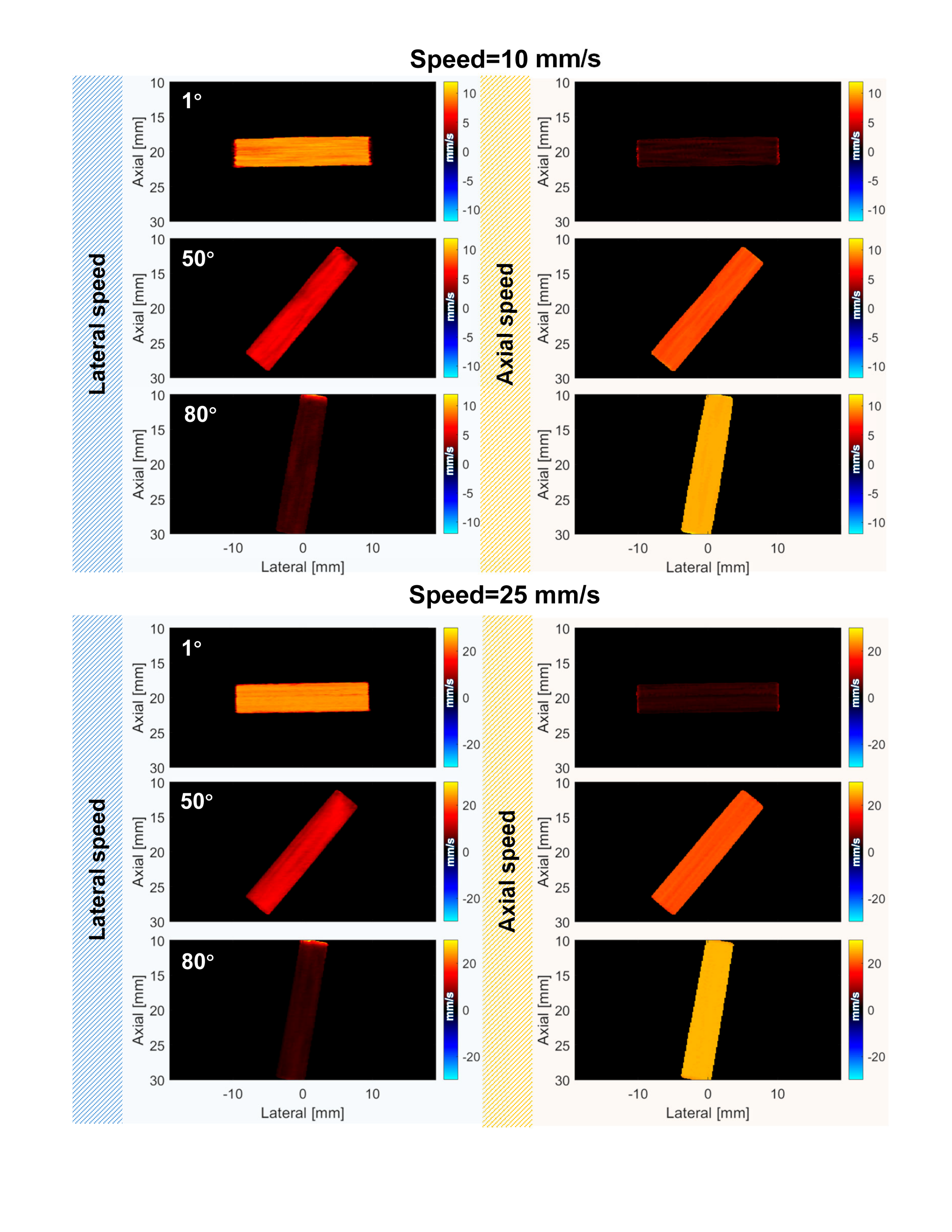
**

**Fig. S3.** The lateral and axial speed maps generated by R-flow under different simulated flow angles and flow speed.


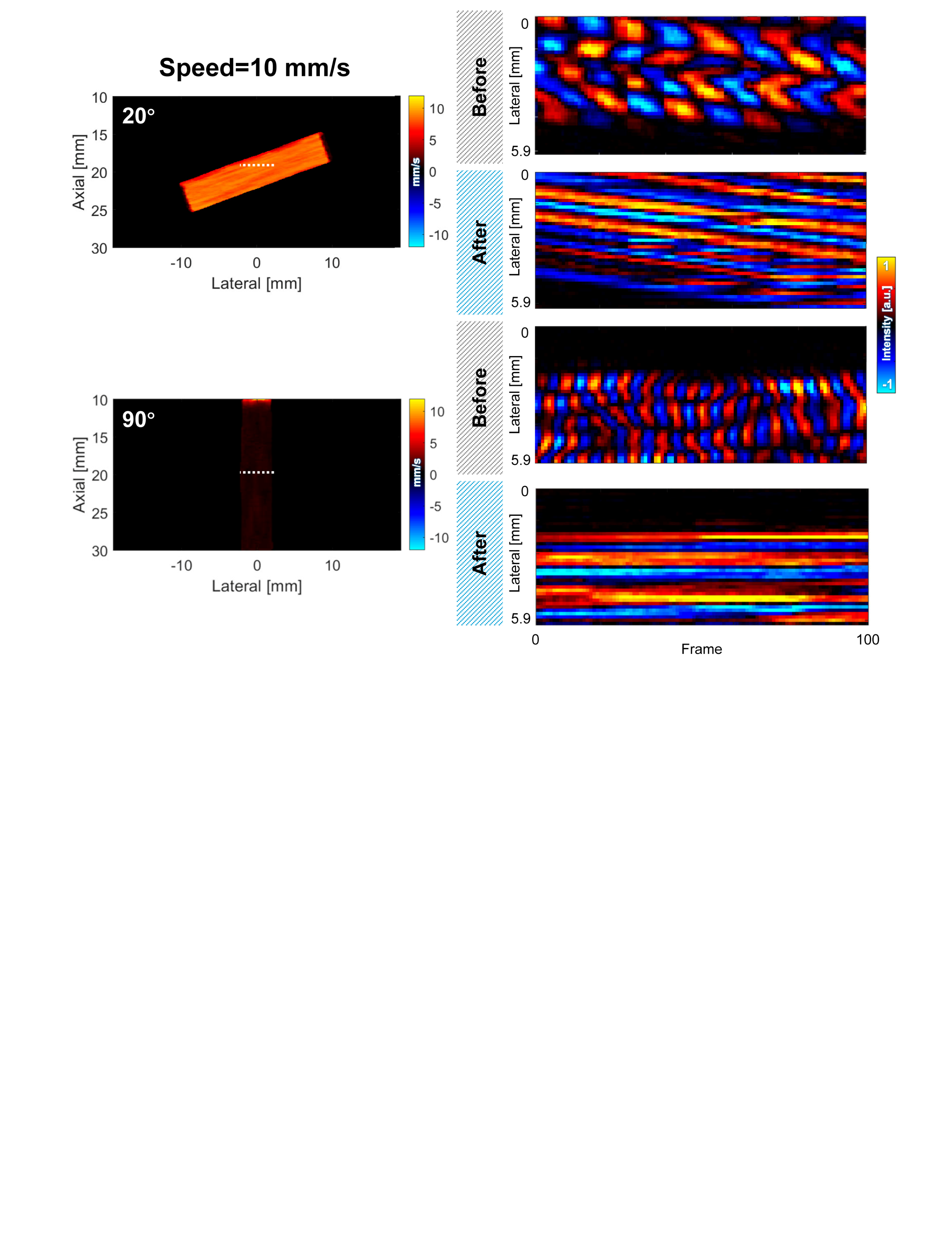


**Fig. S4.** The lateral-temporal image of the IQ signal before and after axial flow motion compensation. Left: lateral velocity maps at 20° and 90° flow angles, with sampling positions marked by white dashed lines. Right: corresponding lateral-temporal images along these lines, showing the real part of the IQ signal before compensation (Before) and the IQ envelope filtered with Sobel-filter after compensation (After).


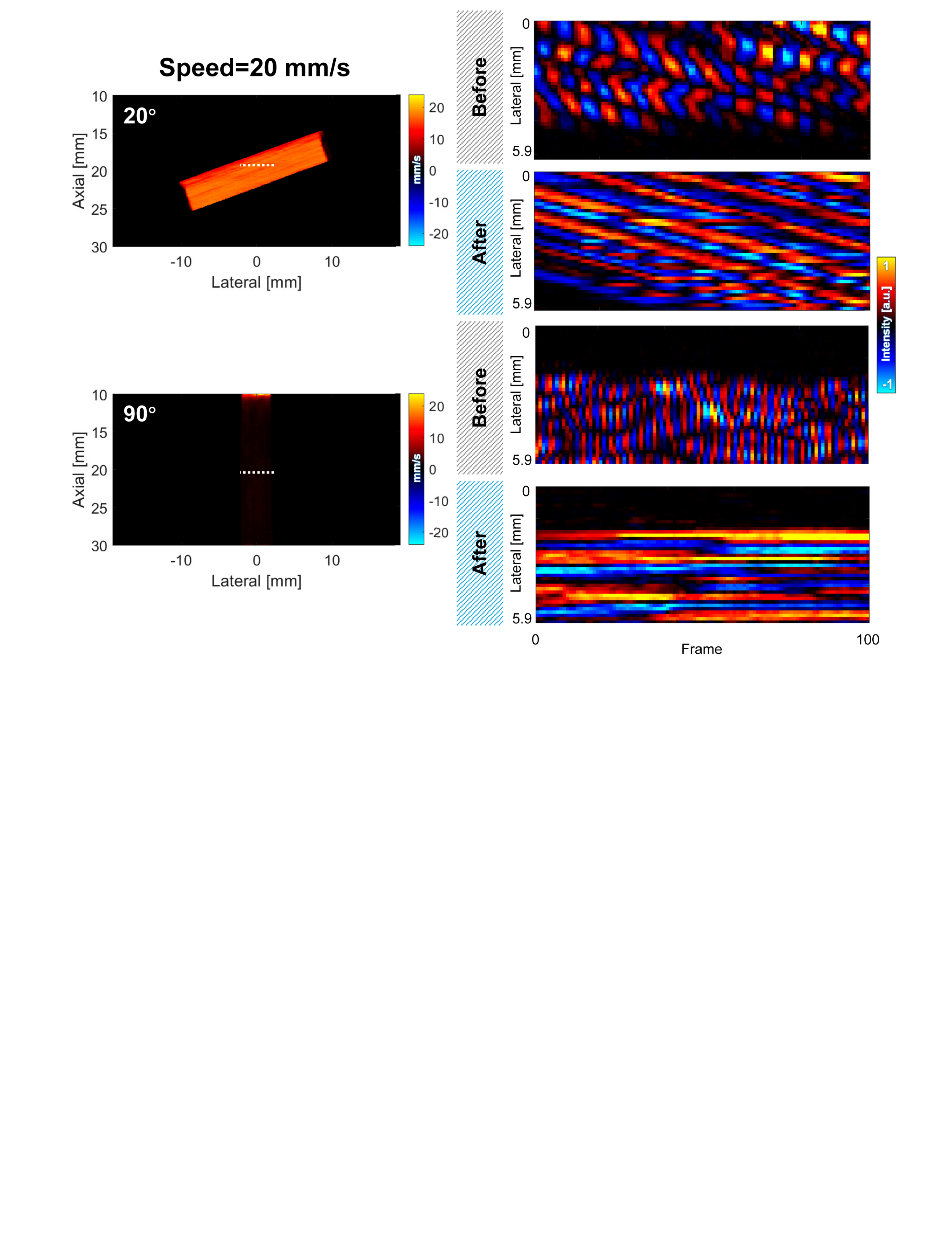


**Fig. S5.** The lateral-temporal image of the IQ signal before and after axial flow motion compensation. Left: lateral velocity maps at 20° and 90° flow angles, with sampling positions marked by white dashed lines. Right: corresponding lateral-temporal images along these lines, showing the real part of the IQ signal before compensation (Before) and the IQ envelope filtered with Sobel-filter after compensation (After).


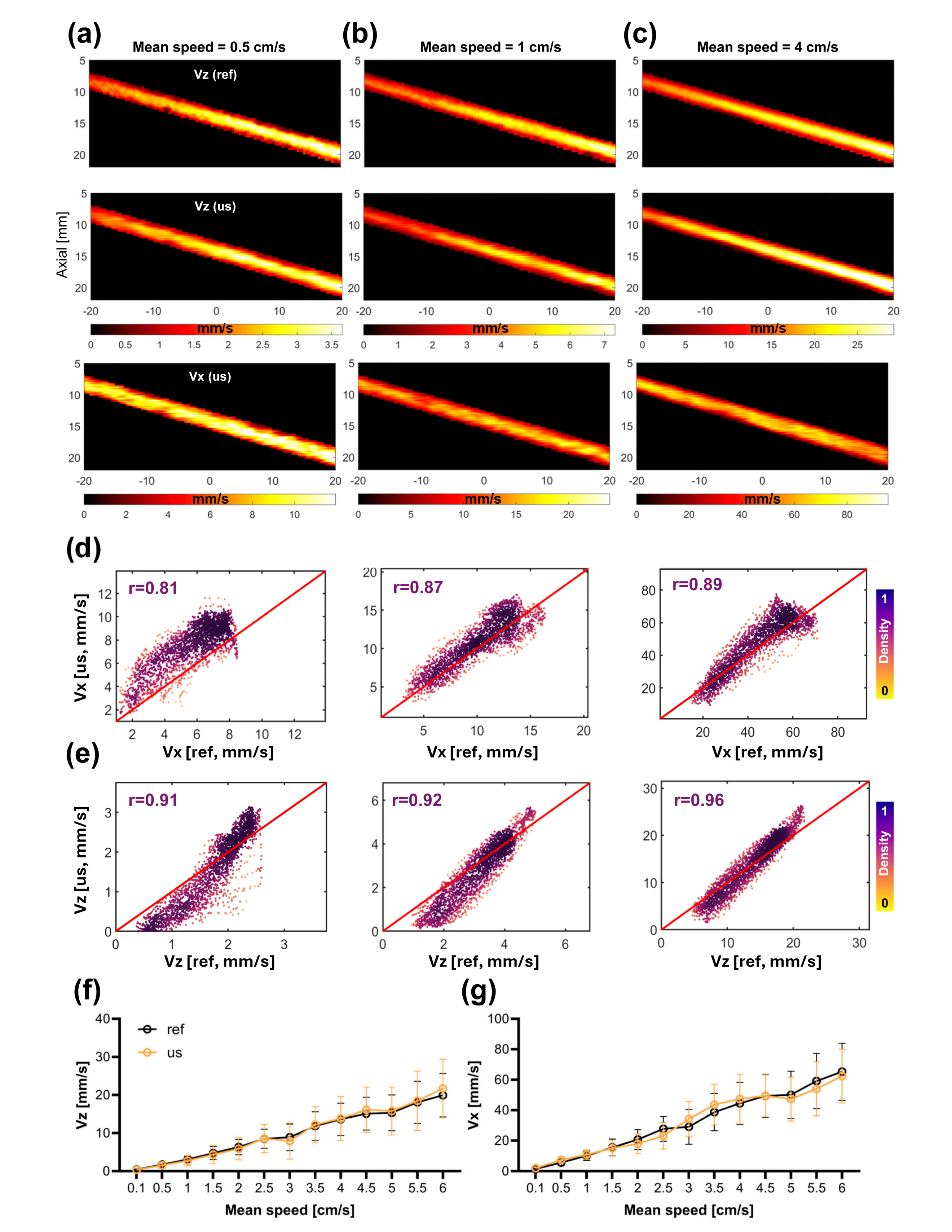


**Fig. S6.** Phantom experiment results. (a–c) Velocity estimation results for mean speeds of 0.5 cm/s, 1 cm/s, and 4 cm/s, respectively. From top to bottom: reference axial speed map *V_z_* derived from Doppler imaging (ref), and axial/lateral speed estimated using R-flow. (d, e) Pixel-wise scatter plots comparing common pixels between R-flow and reference measurements for lateral (d) and axial (e) speed components at three flow speeds (0.5, 1, and 4 cm/s, from the left to the right). The darker regions (deep purple) indicate a higher concentration of matched velocity estimates, and lighter regions (orange or light purple) reflect sparser distributions. The red line indicates the ideal 1:1 ratio, while *r* denotes the Pearson correlation coefficient between the two measurements. (f, g) Lateral (g) and axial speed (f) curves measured with R-flow (us) and reference method (ref) across different flow speeds. Error bars represent standard deviations of measured speed values in the vascular pixels.


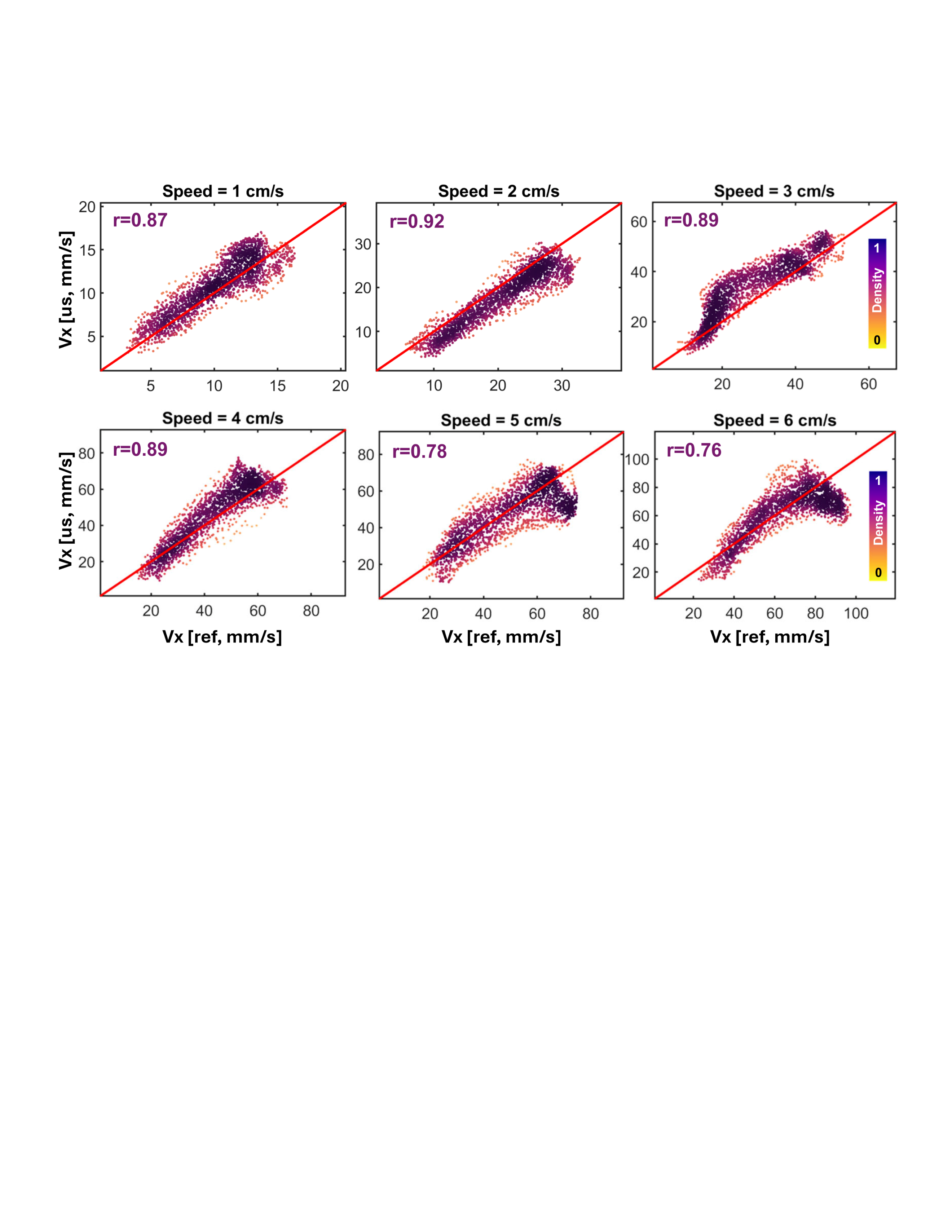


**Fig. S7.** Scatter plots of estimated lateral velocities [*V_x_* (us)] versus reference velocities estimated with Doppler [*V_x_* (ref)] under multiple flow settings in phantom study. The reference lateral speed values were derived from Doppler-based axial velocity estimation and the measured tube inclination angle. Darker color indicates higher density. Each point represents a shared pixel between the estimated and reference maps; the red line indicates the ideal 1:1 line, and *r* denotes the correlation coefficient.


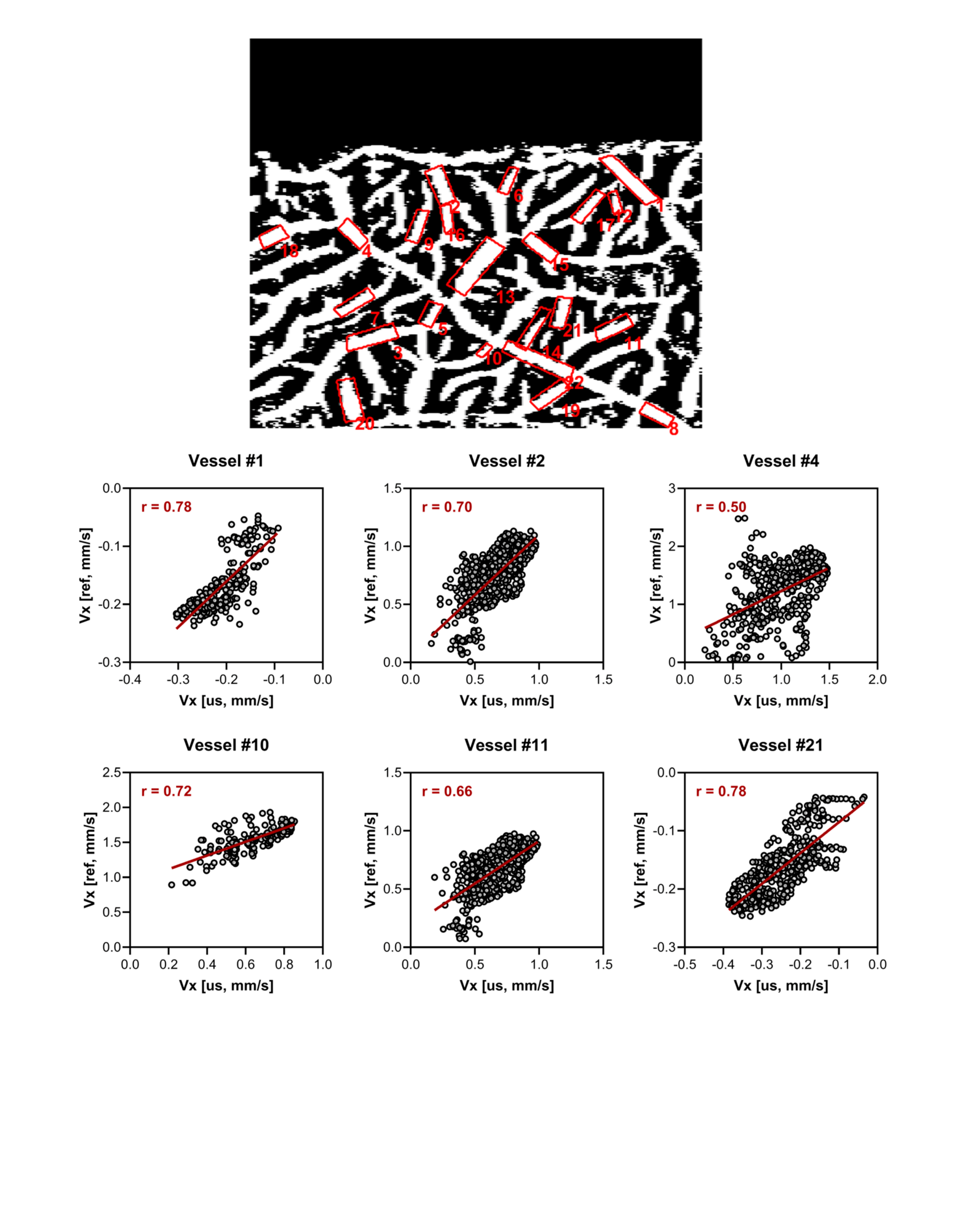


**Fig. S8.** Scatter plots comparing R-Flow estimates of lateral flow velocity [*V_x_* (us)] and reference measurements [*V_x_* (ref)] of chicken embryo data. Each point represents a shared pixel between the estimated and reference maps; the red line indicates the ideal 1:1 fitting line; *r* denotes the correlation coefficient. The location of vessel segments and the number are marked as red rectangles and red text in the binary image of the vessel area.


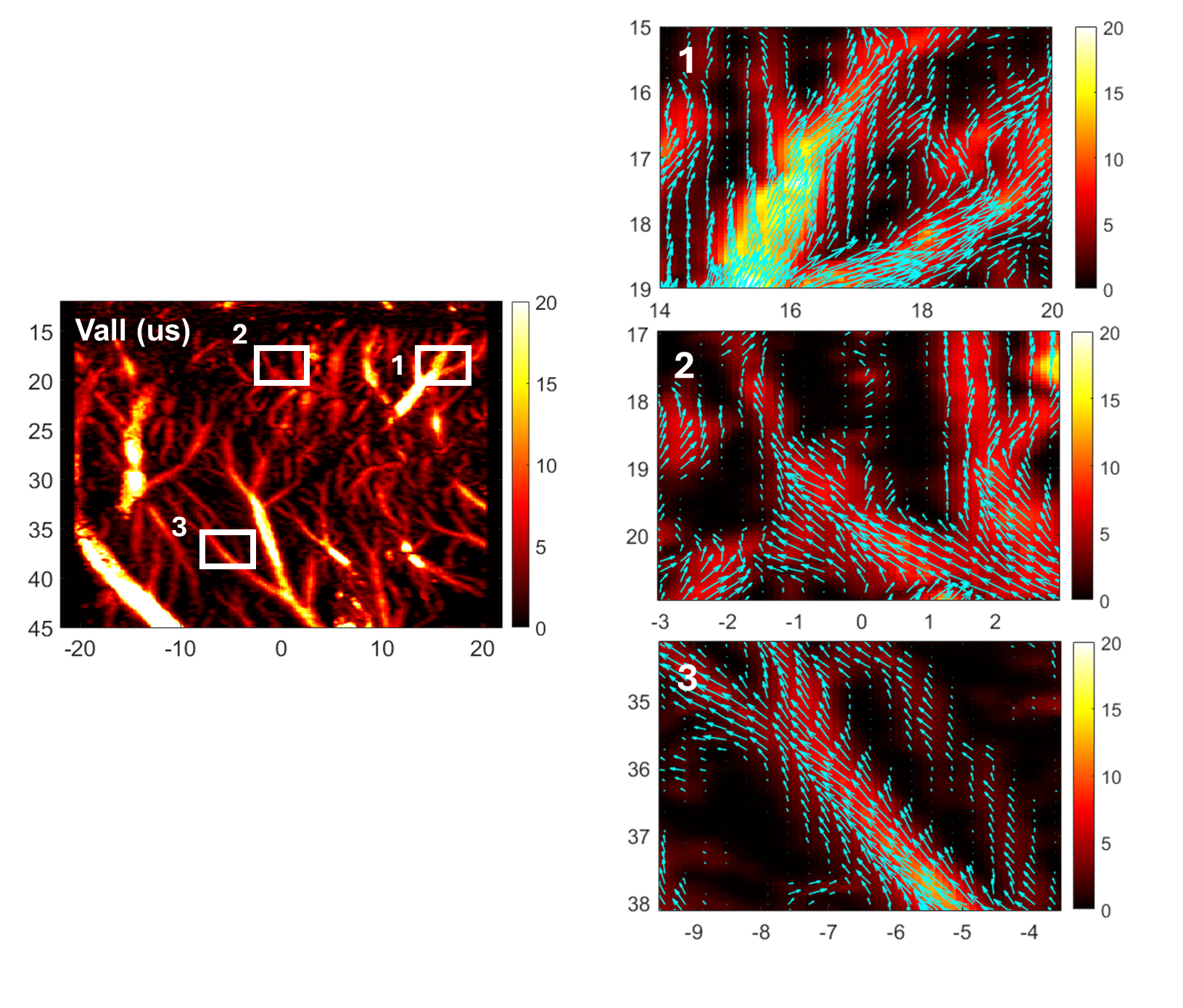


**Fig. S9.** Flow field estimated by R-Flow within representative regions of interest, indicated by white rectangles in speed map (left panel). The local flow direction and speed are visualized using velocity vectors.


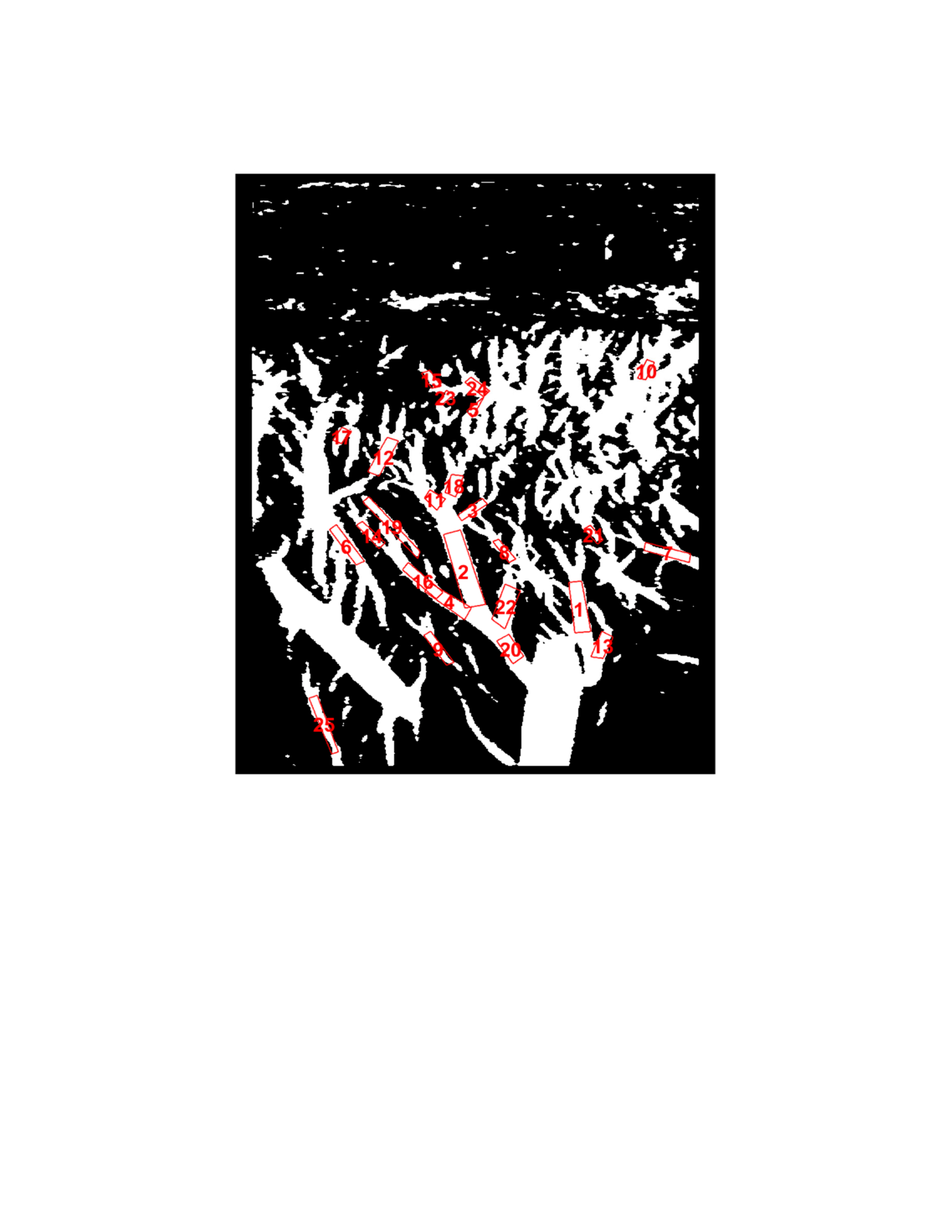


**Fig. S10.** Selected vessel segments for healthy human liver data. The location of vessel segments and the number are marked as red rectangles and red text in the binary image of the vessel area.


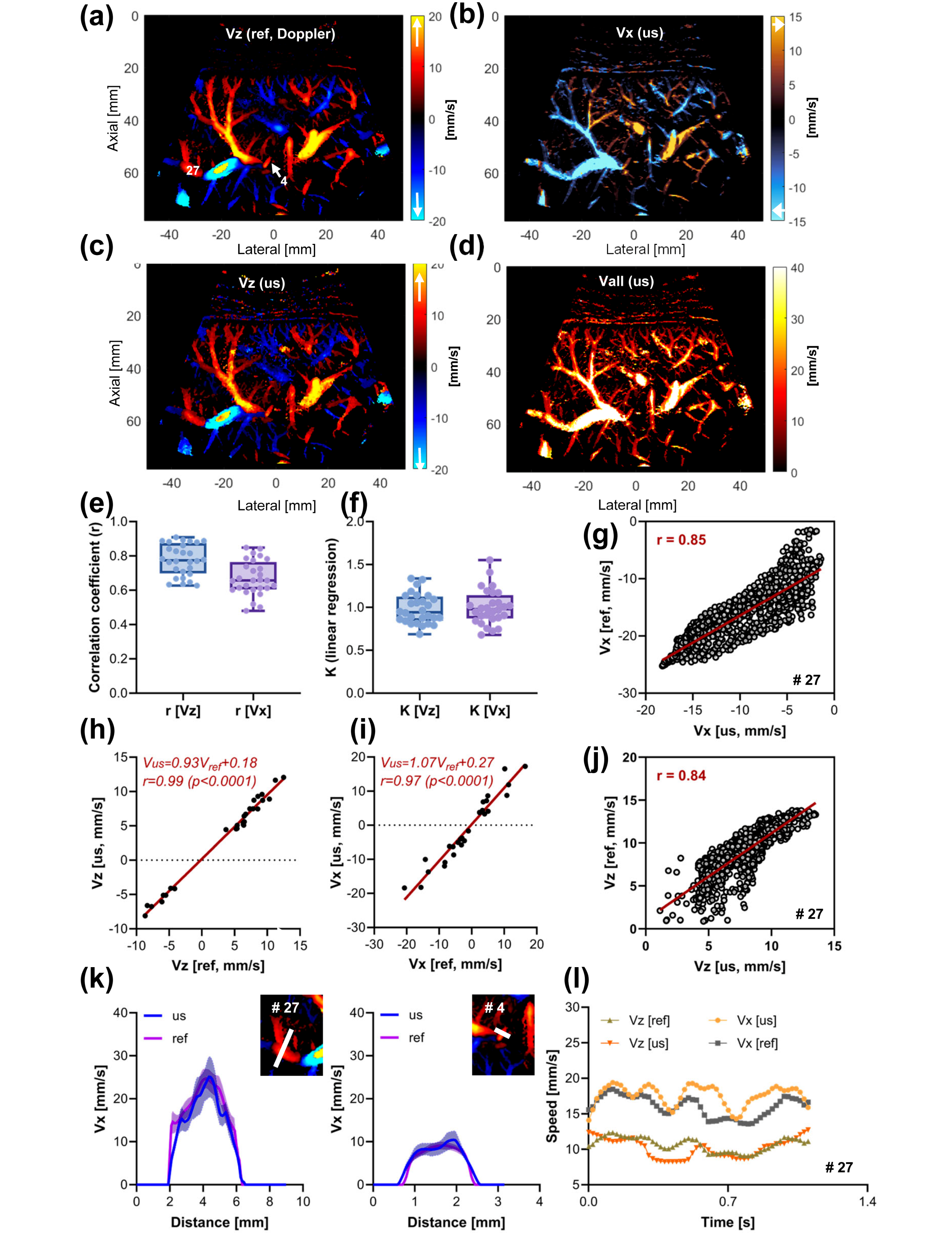


**Fig. S11.** Velocity measurements in a healthy human liver. (**a**) Axial velocity map (*V_z_*) obtained using conventional Doppler as the reference. (**b–d**) Estimated lateral velocity *V_x_* (us), axial velocity *V_z_* (us), and total velocity magnitude *V_all_* (us) using R-Flow. (**e, f**) Pixel-wise correlation coefficients (*r*) and regression slope (*K*) distributions between estimated and reference velocity across 28 representative vessels. *r*[*V_z_*] and *r*[*V_x_*] indicate the correlation coefficients for axial and lateral velocities, while *K*[*V_z_*] and *K*[*V_x_*] represent the regression slopes. (**h, i**) Linear regression between the average lateral (**i**) and axial velocities (**h**) measured by R-Flow and reference method across 28 vessels. (**g, j**) Scatter plots comparing R-Flow estimates (**g**, *V_x_*(us), **j**, *V_z_*(us)) and reference values (ref) at all shared pixels within selected vessels (**Fig. S11**). The red line indicates the ideal 1:1 fitting line; *r* denotes the correlation coefficient. (**k**) Spatial distributions of average lateral flow velocities (along the white line, with vessel position indicated in **a**) and pulsatile velocity ranges across two selected vessel cross-sections #27 and #4. (**l**) Temporal variations of mean lateral and axial velocity components measured at selected vessel locations #27.


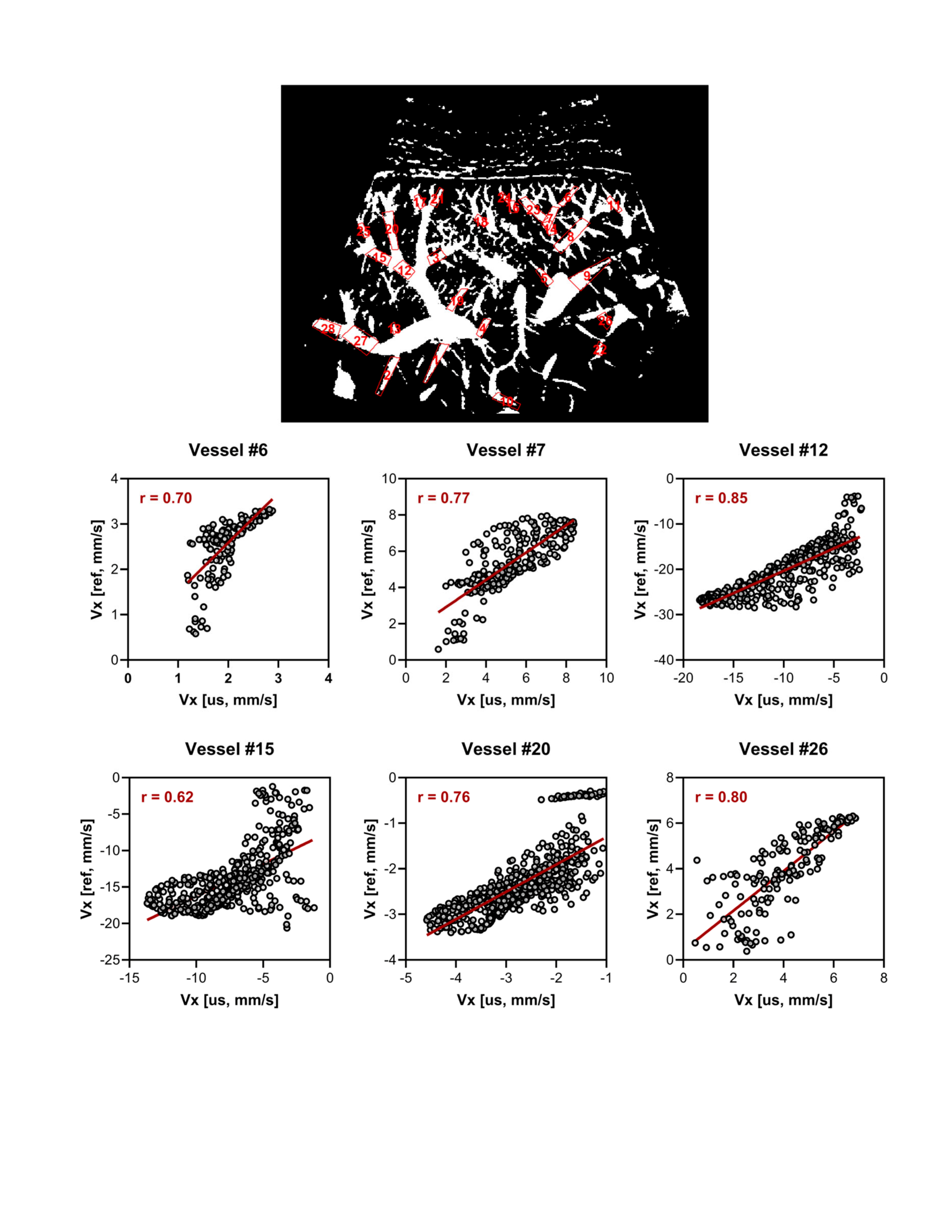


**Fig. S12.** Scatter plots comparing R-Flow estimates of lateral flow velocity [*V_x_* (us)] and reference measurements [*V_x_* (ref)] of human liver data. Each point represents a shared pixel between the estimated and reference maps. The red line indicates the ideal 1:1 fitting line, and *r* denotes the correlation coefficient. The location of vessel segments and the number are marked as red rectangles and red text in the binary image of the vessel area.


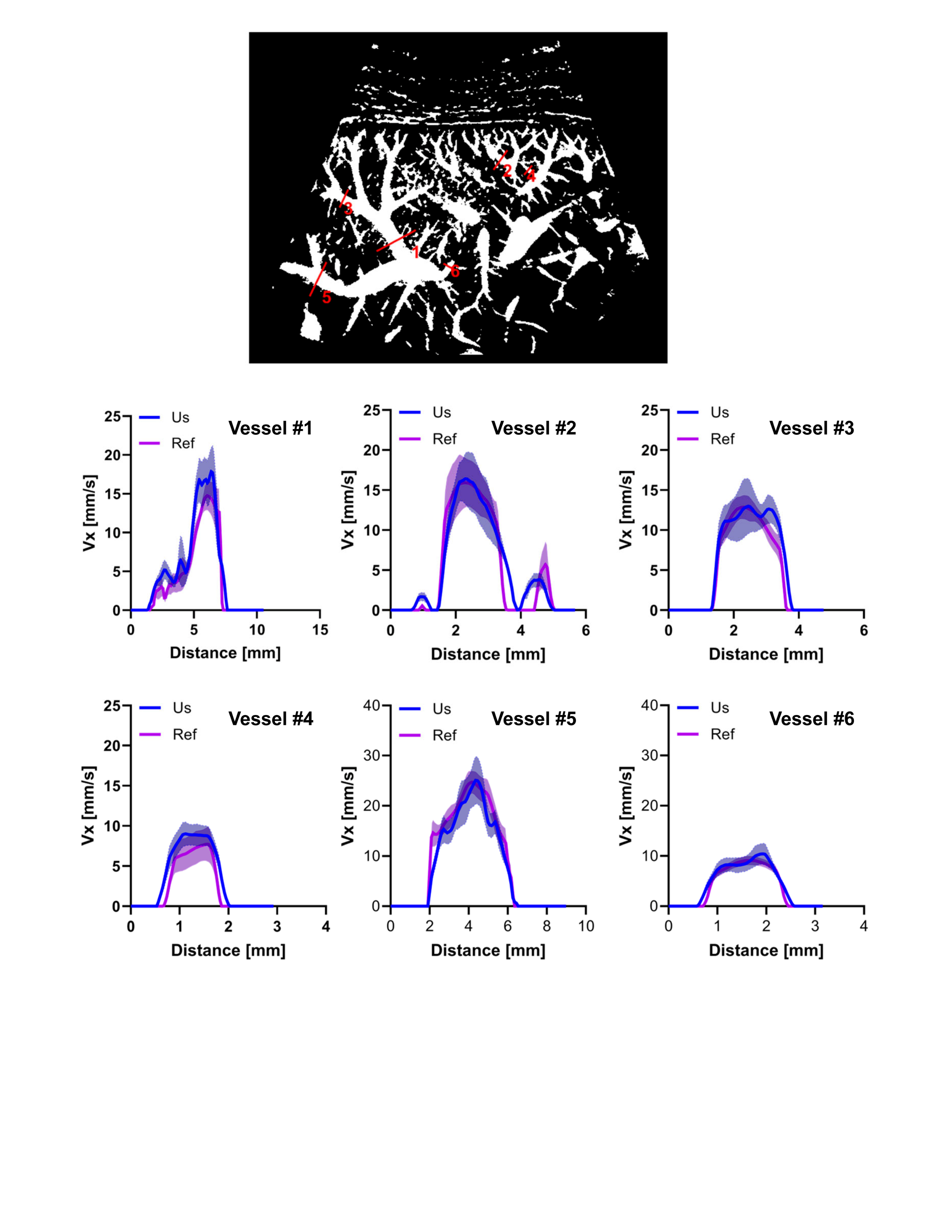


**Fig. S13.** Lateral velocity profiles measured in healthy human liver data. Spatial distributions of average lateral flow velocities (solid lines) and velocity ranges across selected vessel cross-sections. Shading indicates standard deviation across all measurements over time at each location. The location of vessel cross-sections and the number are marked as red lines and red text in the binary image of the vessel area.


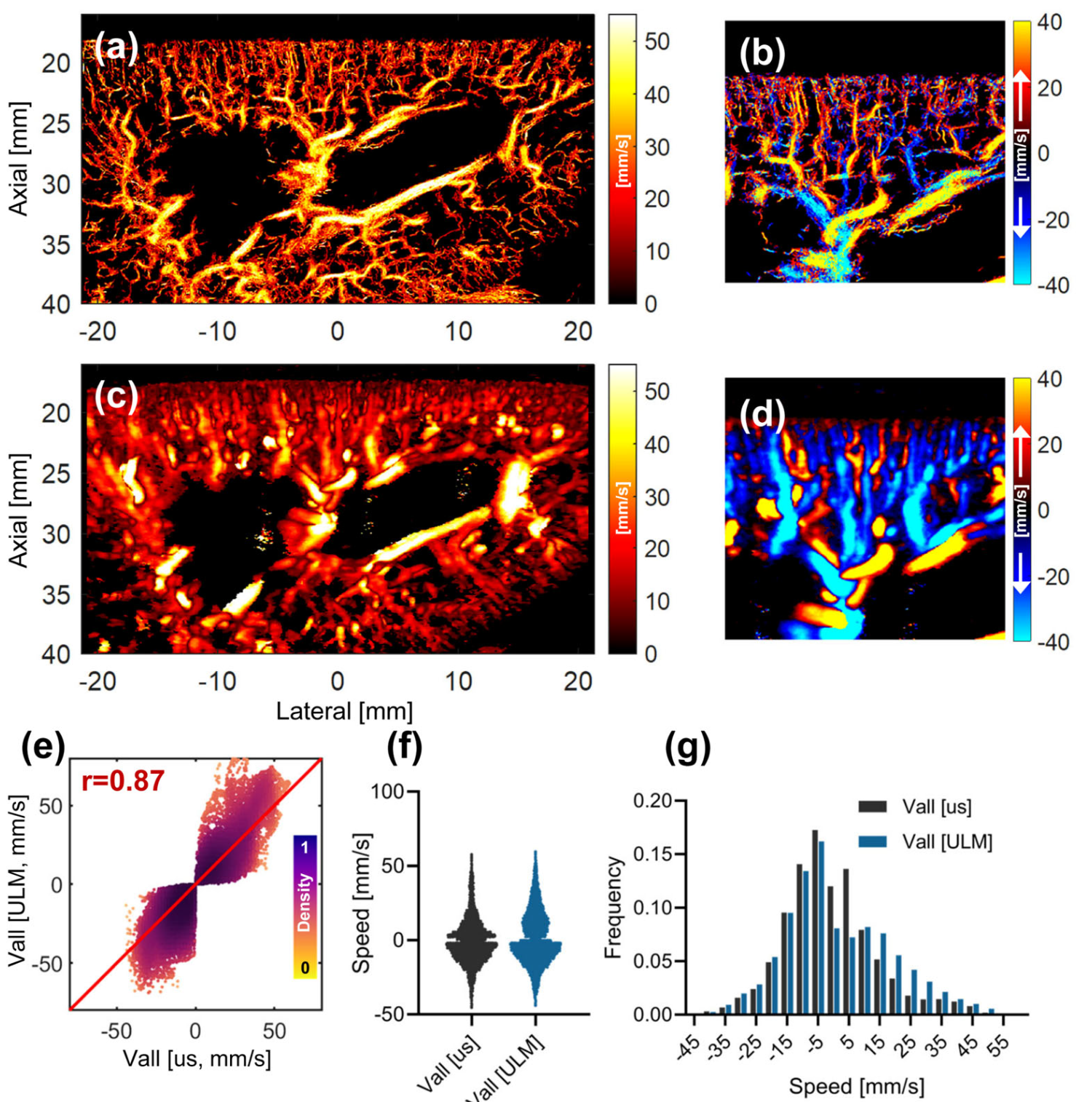


**Fig. S14.** Flow velocity mapping using ULM and R-Flow in a pig kidney. (a, b) Speed map and local axial velocity map measured by ultrasound localization microscopy (ULM). (c, d) Corresponding speed and local axial velocity maps estimated using the proposed R-Flow. (e) Pixel-wise scatter plot comparing flow speeds at matched pixels between ULM (b) and R-Flow (d). The red line indicates the ideal 1:1 ratio, and *r* is the Pearson correlation coefficient. (f, g) Flow velocity distributions from ULM (blue) and R-Flow (black) in the selected areas shown in (b, d), presented as violin plot (f) and histogram (g).


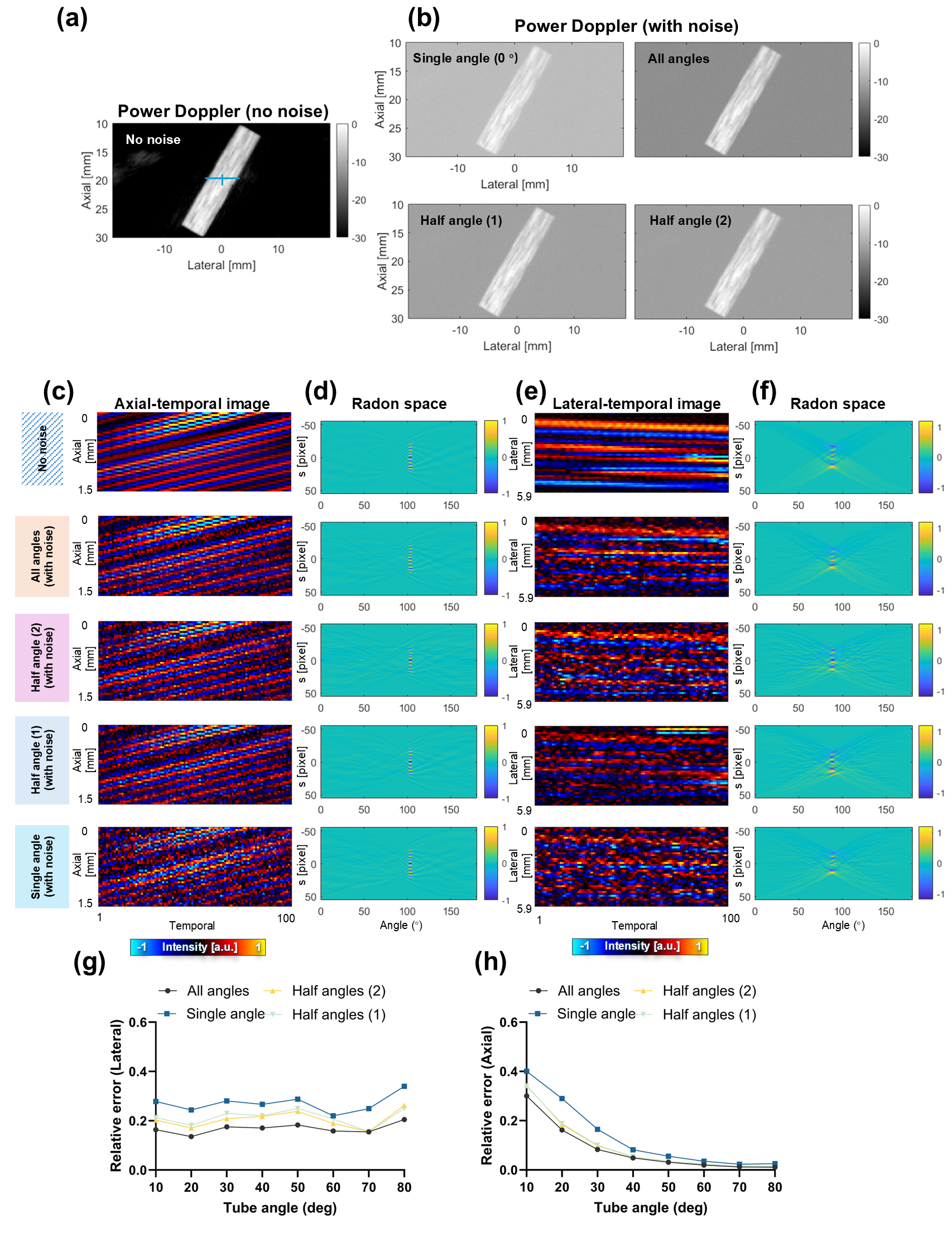


**Fig. S15.** Influence of angular compounding on spatiotemporal trajectories, Radon space, and R-Flow velocity estimation in a plug-flow simulation. (a) Noise-free power Doppler image of a tilted vessel with plug flow (60°), and the blue lines indicate the location used to extract axial–temporal and lateral–temporal signals. (b) Power Doppler images reconstructed from four angular compounding schemes: a single plane-wave angle (0°), two half-angle subsets (half-angle (1): angles #2, #3, and #4; half-angle (2): angles #1, #3, and #5), and full-angle compounding (all five angles). Zero-mean Gaussian noise was independently added to the beamformed RF data of each of the five steering angles, with an SNR of 2 dB defined relative to the average power of the beamformed blood-flow RF signal. (c–f) Axial–temporal (*z*-*t*) images and corresponding Radon spaces (c, d), and lateral–temporal (*x*-*t*) images and corresponding Radon spaces (e, f), for different angular compounding schemes under noisy conditions, with the noise-free axial–temporal case shown on top for reference. (g, h) Relative errors (absolute value) of lateral (g) and axial (h) velocity estimates as a function of tube orientation for the four angular compounding schemes.


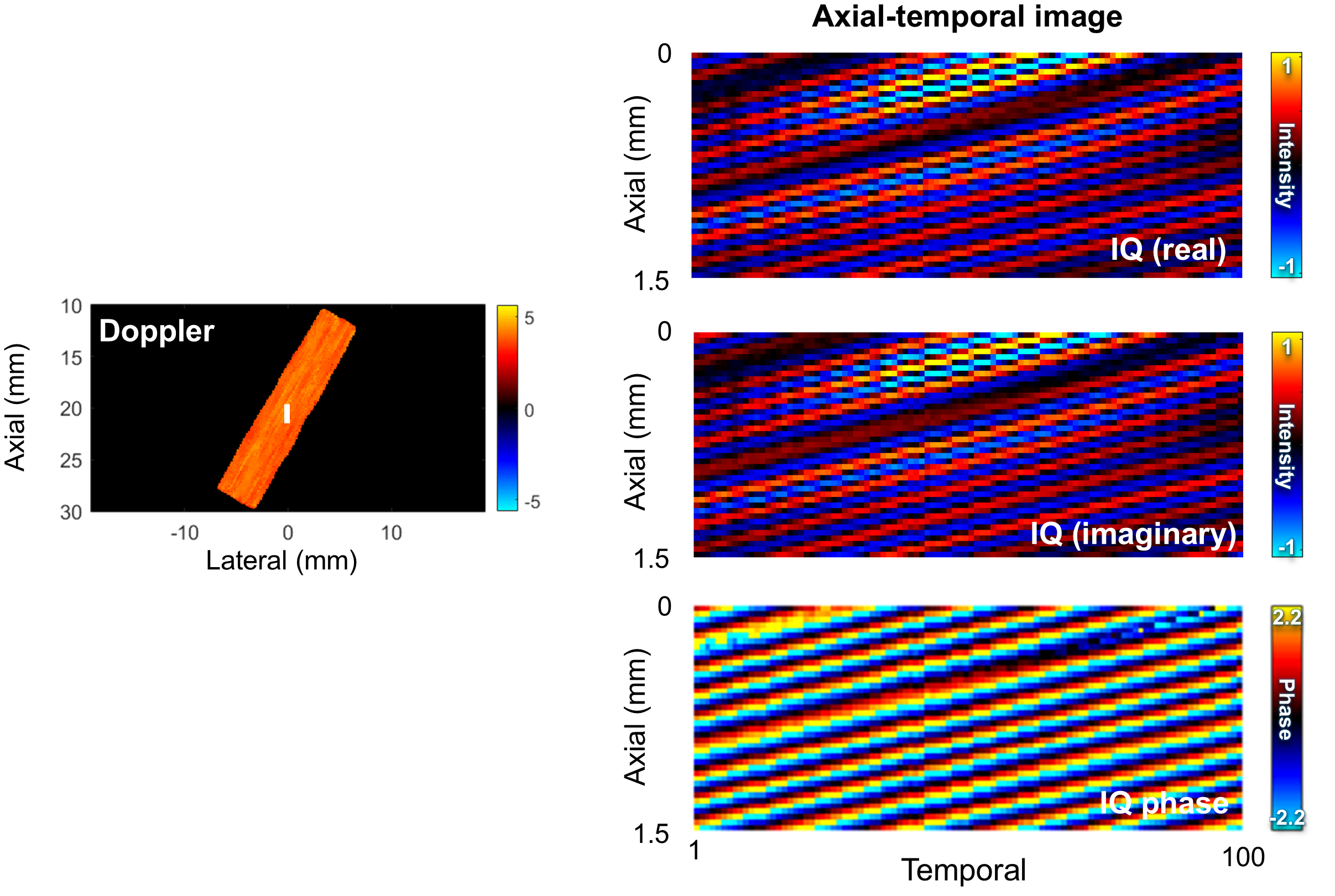


**Fig. S16.** Axial-temporal images of different IQ components. Left: Reference Doppler velocity map of a tilted plug-flow vessel (5 mm/s, 60° tube angle), with the white line indicating the sampled axial position. Right: Axial–temporal (*z*-*t*) spatiotemporal images extracted from the IQ real part, IQ imaginary part, and IQ phase along the sampling line.

### **Supplementary Tables**

**Table S1.** Simulation settings

|  | Value |
| --- | --- |
| Transmit center frequency (MHz) | 5.21 |
| Number of elements | 128 |
| Pitch (mm) | 0.3 |
| Transmission type | Plane wave |
| Steering Angles | −7.5°, −3.75°,0°,3.75°,7.5° |
| Post-compounded frame rate (Hz) | 500 |
| Total number of frames | 300 |
| Number of pulse cycles | 2 |
| Pixel size of IQ  (mm × mm, axial × lateral) | 0.04 × 0.15 |

**Table S2.** Parmeter settings of human liver data

|  | Linear array transducer 9L-D | Curved array transducer C1-6-D |
| --- | --- | --- |
| Transmit center frequency (MHz) | 5.21 | 4.46 |
| Number of elements | 192 | 192 |
| Pitch (mm) | 0.23 | 0.35 |
| Transmission type | Plane wave | Diverging wave |
| Steering Angles | −9° to +9° with 2° increment | −4.5° to +4.5° with 1° increment |
| Post-compounded frame rate (Hz) | 500 | 500 |
| Total number of frames | 300 | 600 |
| Pixel size of IQ  (mm × mm, axial × lateral) | 0.10 × 0.05 | 0.17 × 0.17 |
